# Centromere-Independent Accumulation of Cohesin at Ectopic Heterochromatin Sites Induces Chromosome Stretching during Anaphase

**DOI:** 10.1371/journal.pbio.1001962

**Published:** 2014-10-07

**Authors:** Raquel A. Oliveira, Shaila Kotadia, Alexandra Tavares, Mihailo Mirkovic, Katherine Bowlin, Christian S. Eichinger, Kim Nasmyth, William Sullivan

**Affiliations:** 1 Instituto Gulbenkian de Ciência, Oeiras, Portugal; 2 Department of Biochemistry, University of Oxford, United Kingdom; 3 Department of Molecular, Cell and Developmental Biology, University of California, Santa Cruz, Santa Cruz, California; National Cancer Institute, United States of America

## Abstract

Pericentric heterochromatin, while often considered as “junk” DNA, plays important functions in chromosome biology. It contributes to sister chromatid cohesion, a process mediated by the cohesin complex that ensures proper genome segregation during nuclear division. Long stretches of heterochromatin are almost exclusively placed at centromere-proximal regions but it remains unclear if there is functional (or mechanistic) importance in linking the sites of sister chromatid cohesion to the chromosomal regions that mediate spindle attachment (the centromere). Using engineered chromosomes in *Drosophila melanogaster*, we demonstrate that cohesin enrichment is dictated by the presence of heterochromatin rather than centromere proximity. This preferential accumulation is caused by an enrichment of the cohesin-loading factor (Nipped-B/NIPBL/Scc2) at dense heterochromatic regions. As a result, chromosome translocations containing ectopic pericentric heterochromatin embedded in euchromatin display additional cohesin-dependent constrictions. These ectopic cohesion sites, placed away from the centromere, disjoin abnormally during anaphase and chromosomes exhibit a significant increase in length during anaphase (termed chromatin stretching). These results provide evidence that long stretches of heterochromatin distant from the centromere, as often found in many cancers, are sufficient to induce abnormal accumulation of cohesin at these sites and thereby compromise the fidelity of chromosome segregation.

## Abstract 2

Live imaging of cells carrying rearranged chromosomes shows that misplaced heterochromatin is sufficient to induce ectopic cohesion and chromosome stretching during mitosis, and may compromise genetic stability.

## Author Summary

During cell division, chromosomes acquire their characteristic X-shaped morphology by having well-resolved chromosome arms while still remaining connected at the heterochromatic regions around the centromere. This connection is mediated by the cohesin complex, a “molecular glue” that keeps the two DNA molecules stuck together and ensures that the chromosomes are properly segregated. However, it is unclear how important it is for efficient chromosome segregation that these cohesive forces are specifically positioned near the centromere. In this study, we tested several strains of the fruit fly *Drosophila melanogaster* carrying chromosomal rearrangements in which long stretches of heterochromatin from near the centromere have been misplaced within distant euchromatic regions. We find that such inappropriately located heterochromatin is enough to promote increased levels of cohesin complex loading and the formation of additional constrictions, regardless of proximity to the centromere. Importantly, we further show that as cell division proceeds and the sister chromatids move to opposite poles of the cell, the presence of ectopic heterochromatin (and hence ectopic cohesion) leads to significant chromosome stretching due to impaired resolution of the ectopic cohesion sites. These results highlight the possibility that chromosome rearrangements involving heterochromatin regions near the centromeres, often seen in many cancers, can induce additional errors in cell division and thereby compromise genetic stability.

## Introduction

Although chromosomes contain small segments of heterochromatic regions along chromosome arms, large stretches of heterochromatin containing highly repetitive sequences and spanning several megabases are almost exclusively found around the centromeric region. It is believed that these long stretches of “junk”-DNA result from a cumulative retention due to the inability to recombine DBNA close to the centromeric region [1]. It is also possible that there is a functional significance for retaining extensive heterochromatin regions at centromere-proximal sites while avoiding the presence of dense heterochromatic loci embedded in chromosome arms.

One of the essential functions of the pericentric heterochromatin is to mediate sister chromatid cohesion. Sister chromatid cohesion is brought about by cohesin, a tripartite ring-like protein complex composed of two Structural Maintenance of Chromosome proteins (Smc1 and Smc3) bridged by a kleisin subunit (Rad21/Scc1) [2],[3]. These rings entrap sister chromatids together inside their proteinaceous cage [4]. Chromatid separation is subsequently triggered by proteolytic cleavage of the kleisin subunit by separase [5]–[7]. In metazoa, metaphase chromosomes contain high levels of cohesin solely at the pericentromeric regions [8],[9]. The mechanisms that drive cohesin's accumulation at the pericentromeric regions are not fully understood. Part of this accumulation is known to be due to the Sgo/PP2A-dependent protection mechanism that spares centromeric cohesin from a separase-independent cohesin removal pathway (known as the “prophase pathway”) [10]. This process is mediated by Wapl/Plk and removes most cohesin complexes from chromosome arms during early stages of mitosis [11]–[16].

In addition to the protection mechanisms that maintain cohesin at the pericentromeric region, accumulation of cohesin at these sites might alternatively (or additionally) arise from preferential cohesin loading around the centromere. Whether such accumulation is dictated by the presence of heterochromatin or the centromere has been a matter of debate and may vary according to the organism. In budding yeast, the core centromeres are both necessary and sufficient for cohesin recruitment to neighbouring pericentric sequences [17],[18]. The cohesin loading factor Scc2/4 (NippedB/Mau2) was found to localize preferentially to the centromeres and catalyze loading at these sites during replication [19],[20]. In contrast to the centromere-driven accumulation observed in budding yeast, in fission yeast sister chromatid cohesion is dependent on Swi6 (HP1 homolog) [21],[22]. In metazoa, however, attempts to dissect the link between sister chromatid cohesion and heterochromatin have led to conflicting results. While some studies report mild levels of sister chromatid cohesion defects when the heterochromatic pathway is impaired [23]–[25], others have failed to detect any evident loss of sister chromatid cohesion upon perturbation of pericentric heterochromatin [26],[27]. The exact contribution of heterochromatin to cohesin's enrichment in metazoan chromosomes, therefore, remains unclear.

Pericentromeric accumulation of cohesin is extremely important given that these complexes are the sole counterforce that resists the opposing microtubule pulling forces [7], thereby preventing premature and/or random chromosome segregation. As dense heterochromatin is almost invariably associated with the centromere, it has been difficult to address the exact contribution of heterochromatin and/or centromere proximity to the enrichment of cohesin at pericentric sites. Here we investigate the effect of misplacing pericentromeric heterochromatin at sites distal to the centromere on the recruitment of cohesin and subsequent segregation efficiency during mitosis. Using a series of chromosome rearrangements from *D. melanogaster* we find that ectopic heterochromatin positioned distal to the centromere is sufficient to recruit high levels of cohesin that persist during mitosis. Cohesin is preferentially loaded at heterochromatin regions during interphase due to a high density of cohesin-loading factor Nipped-B at these chromosomal regions. As a consequence, ectopic heterochromatin regions form additional cohesion sites that persist during mitosis. These regions disjoin abnormally during anaphase, resulting in chromatin stretching at the late stages of mitosis. This abnormal chromatin stretching may have severe consequences for the cell to maintain chromosome fidelity.

## Results

### Cohesin Preferentially Associates with Heterochromatin Independent of Its Proximity to the Centromere

**Figure 1:**
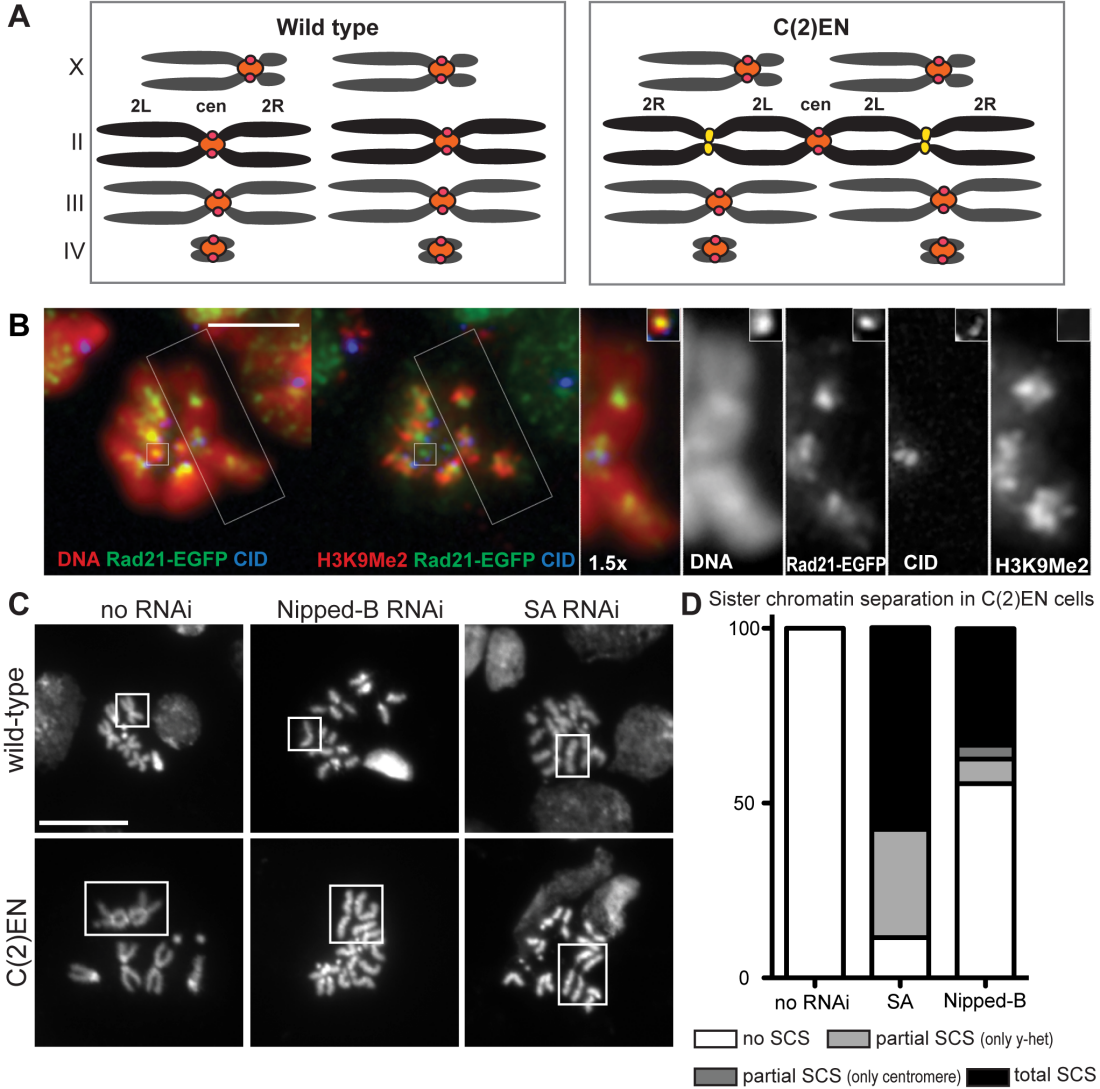
Cohesin regulates constrictions at ectopic heterochromatic sites. (A) Schematic representation of wild-type and C(2)EN karyotypes. Pericentric heterochromatin is labelled in orange, centromeres in red, and ectopic y-heterochromatin in yellow; (B) Spreads from larval brains from C(2)EN strains immunostained for Rad21-EGFP (green) to reveal cohesin localization at the pericentromeric region (centromeres labelled with CID, in blue) and at the displaced heterochromatic (H3diMeK9 labelled red in second left panel). Left panels show a 1.5× magnification of the C (2)EN chromosome and the fourth chromosome (inset) Scale bar is 5 µm; (C) Metaphase spreads after RNAi for the cohesin loader, Nipped-B, and the cohesin subunit, SA, showing premature SCS. Intact 2nd, 3rd, or C(2)EN chromosomes are boxed in the no RNAi control. Corresponding individual sister chromatids resulting from Nipped-B and SA RNAi are boxed in the right panels. Scale bar is 10 µm; (D) Graphical representation of percentage of SCS in C(2)EN cells after SA and Nipped-B RNAi (*n* = 25 for the no RNAi control, *n* = 27 for Nipped-B RNAi, and *n* = 26 for SA RNAi; datasets can be found in Table S2).

In normal chromosomes, cohesin is found almost exclusively at the centromeric region in metaphase, as the Wapl-mediated removal pathway removes most of the cohesin complexes from the chromosome arms [11]–[16],[28],[29]. Whether or not cohesin accumulates and is maintained at heterochromatic regions independent of centromere proximity is currently unknown. To address this, we made use of Drosophila chromosome rearrangements in which large blocks of pericentromeric heterochromatin are embedded in euchromatin and no longer associated with the centromere. For example, in the compound chromosome two, C(2)EN, two homologous arms are connected by a common centromere and each individual arm is linked via Y-heterochromatin [30]. These rearrangements create an unusual metacentric chromosome with arms twice the normal length in which the large stretches of Y-heterochromatin are placed distal to the centromeres by an individual arm's length (Figure 1A). The C(2)EN-bearing stock is euploid and viable.

Previous studies demonstrated that chromosomes containing long stretches of heterochromatin display an ectopic constriction distal to the centromere [31]. In accordance, DAPI staining confirmed that C(2)EN metaphase chromosomes display additional constrictions at the distal heterochromatic regions (Figure 1B). Fluorescent in situ hybridization (FISH) staining with probes specific for repetitive sequences further confirm that the observed constrictions correspond to the ectopic heterochromatin (Figure S1). To monitor cohesin dynamics in these strains we produced transgenic *D. melanogaster* lines expressing a functional version of *rad21* (kleisin subunit of cohesin) tagged with EGFP pan-expressed by the Tubulin promoter. Using a newly developed method to introduce genetic transgenes into compound chromosomes [32], we produced transgenic C(2)EN flies carrying *rad21-EGFP*. This construction allowed us to visualize cohesin localization in these strains, both by live cell imaging and immunofluorescence (Figure 1B). Importantly, metaphase spreads of C(2)EN neuroblast cells revealed high levels of Rad21-EGFP at the ectopic heterochromatic sites (Figure 1B). These regions are also positively labelled with heterochromatin specific markers, such as histone 3 diMeK9, but lack a proximal centromere, as determined by the absence of CID staining (Figure 1B). Cohesin localization is very similar to diMeK9 (although more restricted to the internal part of the chromosome), with the exception of the small Chromosome IV, which contains high levels of cohesin although little diMeK9 could be detected (Figure 1B, insets).

To determine if these results are a general property of displaced pericentric heterochromatin, we examined the *T(2;3)lt^x13^* translocation, a rearrangement in which a large block of pericentric heterochromatin is separated from the centromere [33]. As found in the compound chromosome, Rad21-EGFP localizes at the displaced heterochromatin site. In contrast, an inversion bearing only euchromatin breakpoints (In(3LR)264) does not produce ectopic cohesin localization (Figure S2).

The strong cohesin accumulation at ectopic heterochromatin sites argues that the detected constrictions are mediated by cohesin. To test this notion we depleted a cohesin subunit (stromalin [SA]) or a cohesin loading protein (the Drosophila Scc2 ortholog, Nipped-B). Using the central nervous system specific driver, ElaV-Gal4, we drove UAS-TRiP RNA interference (RNAi) lines in wild-type and C(2)EN-bearing larval brains. As expected, RNAi in wild-type cells resulted in premature sister chromatid separation (SCS) (Figure 1C, top panel). In support of cohesin-mediated ectopic pairing, RNAi for SA and Nipped-B revealed loss of sister chromatid cohesion along the entire chromosome length, including ectopic pairing sites in C(2)EN chromosomes (Figure 1C, bottom panel). In the majority of C(2)EN neuroblast cells expressing the SA RNAi line, premature SCS in metaphase was observed at both the ectopic Y-heterochromatic sites and the centromere (57.7% of cells, *n* = 26) (Figure 1D). The Nipped-B RNAi was less effective as both control and C(2)EN cells show a high percentage of cells with no premature SCS (Figure 1D and unpublished data). Nevertheless, in C(2)EN cells expressing Nipped-B RNAi a high frequency of cells display loss of cohesion along the entire length of the chromosome (33.3% of cells, *n* = 27). Partial SCS (solely at the Y-heterochromatic sites) was also observed in a portion of the cells, however at a lower frequency (30.8% of cells in SA RNAi and 7.4% of cells in Nipped-B RNAi). On the basis of these results we conclude that cohesin is responsible for the tightly conjoined ectopic heterochromatic regions.

### Cohesin Is Preferentially Loaded at Pericentromeric Regions

**Figure 2:**
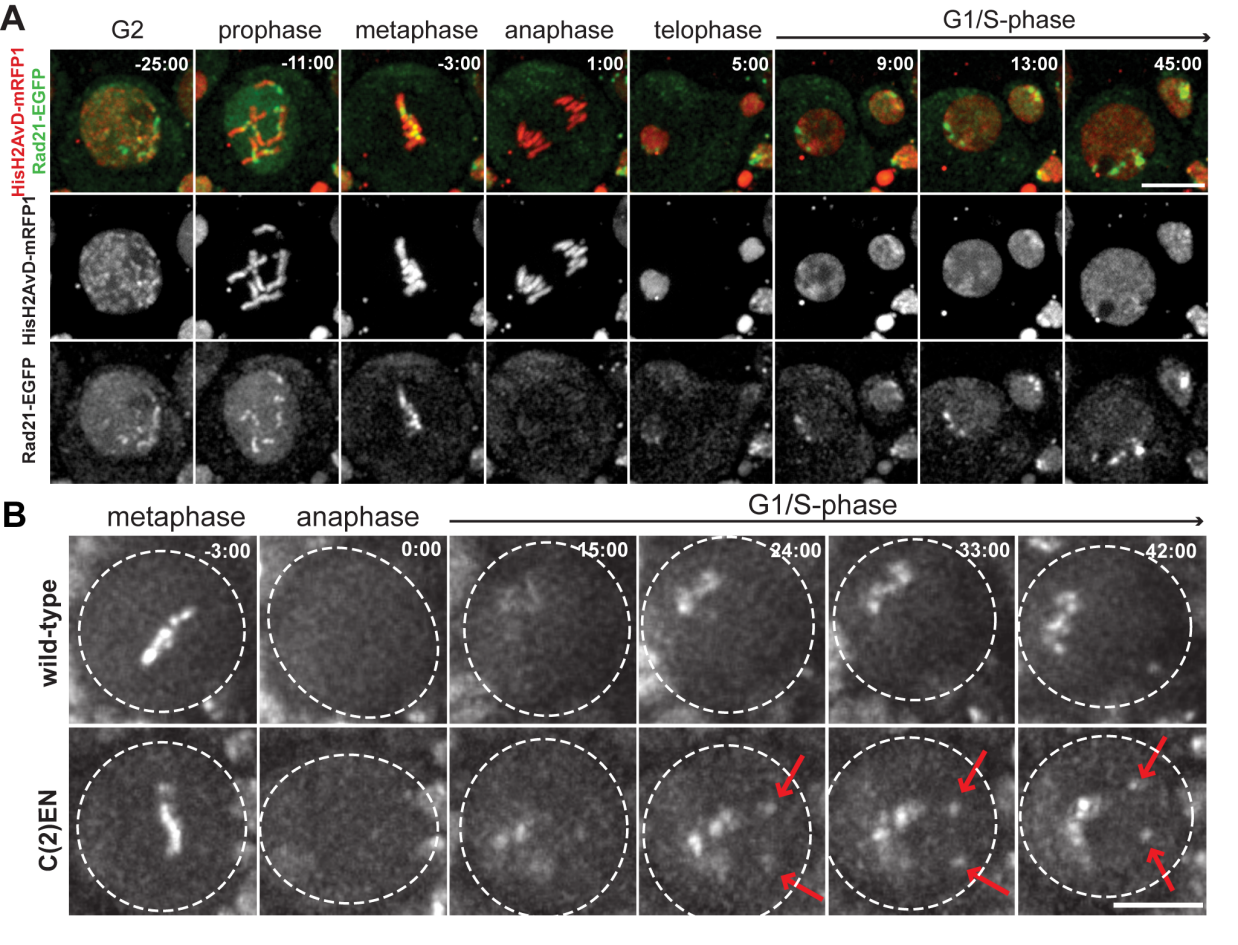
Live imaging reveals that cohesin is enriched at heterochromatic regions during G1/early S-phase. (A) Drosophila larval neuroblasts cells containing Rad21-EGFP (green) and HisH2AvD-mRFP1 (red) display distinct Rad21 foci in early interphase prior to or during S-phase; (B) Stills from live-cell imaging of Rad21-EGFP in wild-type and C(2)EN neuroblast cells. Note that shortly after mitotic exit, a strong accumulation of Rad21-EGFP is detected at the pericentromeric region in wild-type cells and at two additional foci (arrows) in C(2)EN. Times 0∶00 equals anaphase onset. Scale bars are 10 µm.

The results above suggest that cohesin accumulation is determined by the presence of heterochromatin rather than centromere proximity. To dissect when such accumulation is first observed relative to the cell cycle, we performed time-lapse microscopy in strains carrying Rad21-EGFP. In *Drosophila* in larval neuroblasts, we found that most cohesin complexes associated with chromatin before or very early in S-phase (note in this cell type G1 is very short and S-phase follows almost immediately after the previous mitosis) (Figure 2; Movie S1). Importantly, at this time, a strong accumulation of cohesin could be detected specifically at the pericentromeric regions (Figure 2A and 2B). Quantitative analysis of His-mRFP levels reveals that pericentric heterochromatin contains 1.45-fold more histones than euchromatin sites (Figure S3A). Rad21-EGFP, in turn, is 2.4-fold more abundant at the pericentromeric regions when compared to places distal to the centromeres. Therefore, the high levels of cohesin detected at pericentromeric regions are not simply due to increased chromatin compaction. Flies expressing both Rad21-EGFP and Cid-mRFP further confirm that cohesin foci are detected very early during S-phase in regions very close (but not co-localizing) with the centromeres (Figure S4). Moreover, in contrast to wild-type cells where we mostly detect cohesin enrichment at the pericentromeric cluster, C(2)EN chromosomes contain two additional foci appearing during S-phase at sites distal to the clustered centromeres (Figure 2C, arrows; Movie S2). These distal sites are often close to chromatin-dense regions (Figure S3B), similar to what was found for these ectopic heterochromatin regions (Figure S1). Moreover, live cell imaging of HP1-EGFP in C(2)EN strains show a localization pattern resembling Rad21-EGFP (Figure S5). These findings reveal that cohesin localizes to heterochromatin-dense regions (both centromere-proximal and ectopic) very early in the cell cycle (before or early S-phase).

**Figure 3:**
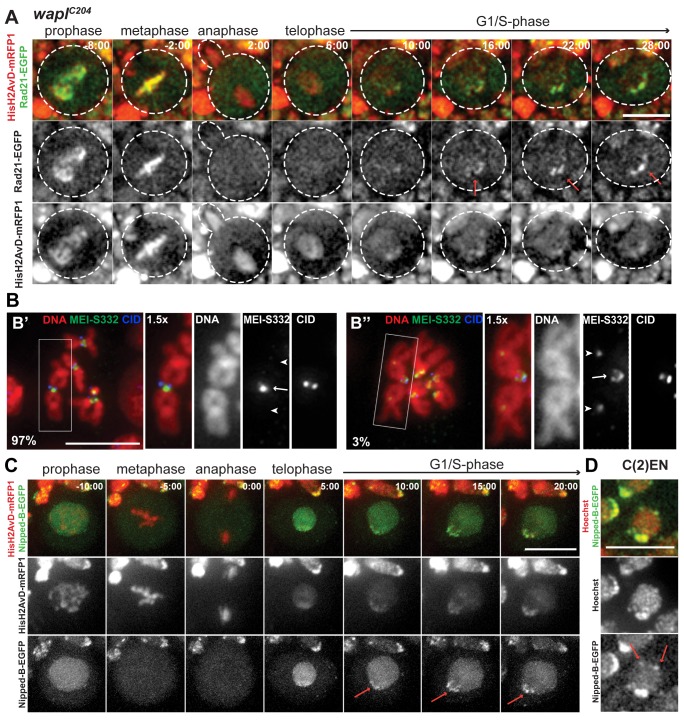
Cohesin enrichment at pericentromeric regions results from preferential loading. (A) Images from live analysis of larval neuroblasts in a *wapl^C204^* mutant background (Rad21-EGFP [green] and HisH2AvD-mRFP1 [red]). Note that Rad21-EGFP is still enriched at the pericentromeric regions (arrows). Time 0∶00 equals anaphase onset; (B) Spreads from larval brains from a C(2)EN strains immunostained for MEI-S332/Shugoshin (green) and CID (blue). DNA is shown in red. Percentages indicate the frequency of cells without (B′) and with (B′′) detectable MEI-S332 staining. In the vast majority of the cells, MEI-S332/Shugoshin is found at the pericentromeric regions (arrow) but not at the ectopic heterochromatin (arrow-heads); (C) Images from live analysis of Nipped-B-EGFP (green) in wild-type Drosophila neuroblasts demonstrate enrichment of this cohesin loader at heterochromatic regions during S-phase (arrows). DNA is labelled with HisH2AvD-mRFP1 (red). Time 0∶00 equals anaphase onset; (D) Image from live analysis of Nipped-B-EGFP (green) in C(2)EN-bearing Drosophila neuroblasts demonstrates enrichment at two additional foci distal to the pericentromeric cluster (arrows). DNA is labelled with Hoechst (red). Scale bars are 10 µm and apply to all images.

The high accumulation of cohesin at the heterochromatic regions could in principle result from two different mechanisms. Either cohesin is loaded preferentially at the pericentric sites, or cohesin associates with chromatin equally throughout the entire chromosome length but is then selectively removed at regions distal to the centromeres. To distinguish between these two scenarios we first analysed cohesin loading dynamics in a Wapl mutant background (*wapl^C204^*), a probable null allele known to have a defective prophase pathway [34]. Our recent studies have indicated that Wapl is involved in cohesin's removal from chromatin by opening the interface between Smc3 and Rad21 [16]. Importantly, this activity is not only present during early mitosis but also throughout the entire cell cycle and in non-dividing cells, and ensures turn-over of cohesin at chromosome arms [16]. If cohesin's accumulation at pericentric sites stems from an increased release of cohesin around chromosome arms, we would expect that inhibiting such a release pathway should abolish preferential accumulation of cohesin at centromeres. However, even when Wapl is mutated, and thereby cohesin removal at chromosome arms is impaired, we are still able to detect a stronger accumulation of cohesin at the pericentromeric regions versus the distal chromosome arms during early S-phase (Figure 3A; Movie S3).

As the high density of cohesin at ectopic heterochromatin sites persists during mitosis, we next addressed if this accumulation was caused by the localization of the cohesin protector MEI-S332 (the *Drosophila* ortholog for Sgo1), a protein required for maintenance of cohesin at the centromere [35]. Squashes of colchicine-arrested neuroblasts reveal that during metaphase, the putative cohesin protection protein MEI-S332 is detected at the inner-centromeres but not at the ectopic heterochromatin sites (Figure 3B). While cohesin is detected in similar amounts between the pericentric and the ectopic heterochromatin stretches of C(2)EN, almost no MEI-S332 could be detected at the distal ectopic sites (only 1/30 cells displayed very reduced levels of MEI-S332). Although we cannot formally exclude that residual (undetectable) levels of MEI-S332 could be responsible for protection, our results suggest that cohesin accumulation at ectopic heterochromatic sites is independent of MEI-S332.

**Figure 4:**
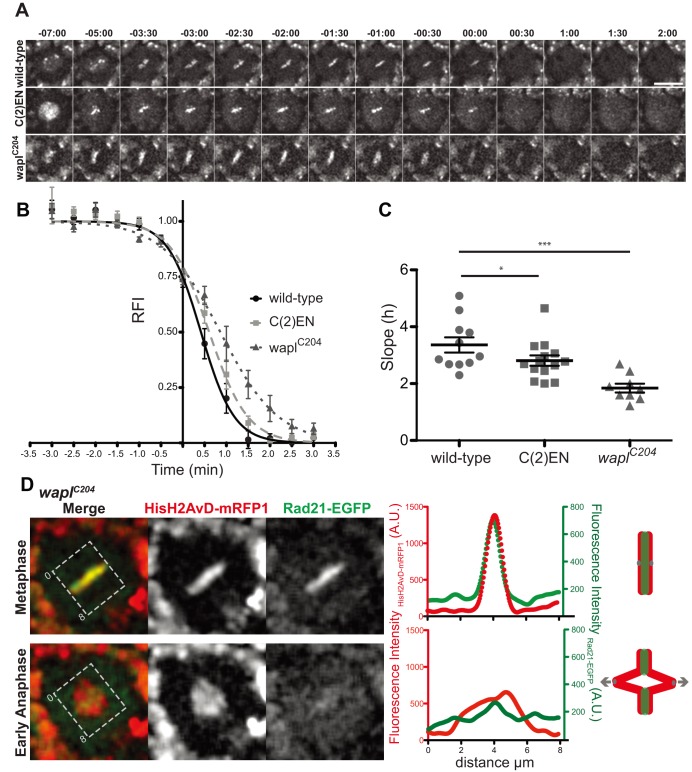
Cohesin degradation is delayed in C(2)EN and Wapl mutants. (A) Images from live analysis of Rad21-EGFP dynamics in wild-type, C(2)EN, and *wapl^C204^* mutant neuroblast cells. Time 0∶00 equals anaphase onset and scale bars are 10 µm. (B) Relative fluorescence intensity (RFI) of Rad21-EGFP over time; levels were normalized to the time of mid-metaphase (3–4 min before anaphase); different movies were aligned to the anaphase timing, defined as the time where Rad21-EGFP levels have dropped below 85% (RFI <0.85). Each data point represents the average ± (standard error of the mean) SEM (datasets can be found in Table S2). A sigmoidal curve was used to fit the data; (C) Graphic representation of the slope of the sigmoidal curve (h). C(2)EN and *wapl^C204^* are significantly different from the controls (*n*≥10 for each condition, *p<0.05; ***p<0.0001, one-tailed students *t*-test); (D) Analysis of spatial distribution of Rad21-EGFP signal (green) during metaphase and early anaphase. DNA is labelled in red. Left intensity profiles were obtained by drawing a box, parallel to the segregation axis. Note that after anaphase onset, a significant peak of Rad21-EGFP signal can still be detected at the regions that lag behind the chromatin mass.

The above results indicate that cohesin accumulation at pericentromeric regions does not stem from protection from the releasing mechanisms and suggest that instead, loading of cohesin throughout S-phase may be particularly enhanced at these chromosomal loci. To test this hypothesis we have evaluated the differential localization of the cohesin-loading factor, Nipped-B, at different chromosomal regions during cell division. Using Drosophila lines expressing functional versions of Nipped-B-EGFP [36], we have found that the cohesin loader strongly accumulates at the centromere-proximal regions throughout S-phase, but is absent upon mitotic entry in wild-type neuroblasts (Figure 3C; Movie S4). Similar to cohesin (Rad21-EGFP), Nipped-B foci also appear at sites near, but not colocalizing with CID (Figure S4B). Importantly, a strong accumulation of NippedB-EGFP could also be detected at two additional foci, distal to the pericentromeric cluster, in C(2)EN chromosomes (Figure 4D). Taken together, these results indicate that cohesin enrichment at pericentromeric regions results from a preferential loading at these sites rather than selective removal along chromosome arms.

### Ectopic Cohesin Induces a Slight Delay in Cohesin Cleavage at Metaphase-Anaphase Transition

Our results show that high levels of cohesin associated with ectopic heterochromatin sites that persist through metaphase. To evaluate whether this additional cohesion influences the dynamics of chromosome segregation we tested whether it affects cohesin's removal from chromosomes. We analysed quantitatively the disappearance of cohesin at the metaphase-anaphase transition from both wild-type and C(2)EN chromosomes. Images were collected every 30 seconds and the levels of Rad21-EGFP monitored over time (Figure 4). To quantify the kinetics of cohesin degradation, data points were fit to a sigmoid curve and the efficiency of its removal was inferred from the slope (h) of the sigmoid curve (see Materials and Methods). This analysis revealed that cohesin disappears from chromosomes with slightly slower kinetics in the C(2)EN strain (h = 3.36±0.27 in controls compared to 2.87±0.16 in C(2)EN). These results indicate that the presence of ectopic cohesion at sites distal to the centromeres delays cohesin removal from chromosomes (Figure 4B and 4C). To further confirm this notion we evaluated the dynamics of cohesin cleavage in strains where the removal of cohesin along chromosome arms is impaired. In *wapl^C204^* cells chromosomes display a high level of cohesin along the entire chromosome arms, which are then cleaved by separase at the metaphase to anaphase transition. Analysis of Rad21 removal dynamics in *wapl^C204^* strains reveals that the presence of cohesin along the entire chromosome length leads to a pronounced delay in cohesin removal (h = 1.84±0.16) (Figure 4B and 4C). This delay can either be caused by exceeded separase cleavage capacity or, alternatively, by a less efficient cohesin removal at chromosome arms. To distinguish between these two possibilities we have analysed the spatial distribution of cohesin in very early anaphase cells in *wapl^C204^* mutants. Limiting cleavage capacity should lead to a delay in cohesin removal that is homogeneous along the chromatin mass. However, we observe that in early anaphase cells, while separated chromatin regions (centromere-proximal) are devoid of Rad21, chromatin placed in the middle of the segregation plate (chromosome arms) still accumulate detectable levels of Rad21 (Figure 4D). Although we cannot formally exclude that separase is rate-limiting in these cells, these results support that cohesin removal at sites distal to the centromere is delayed relative to centromere-proximal regions.

### Chromosomes with Large Portions of Heterochromatin Distal to the Centromere Stretch during Anaphase

**Figure 5:**
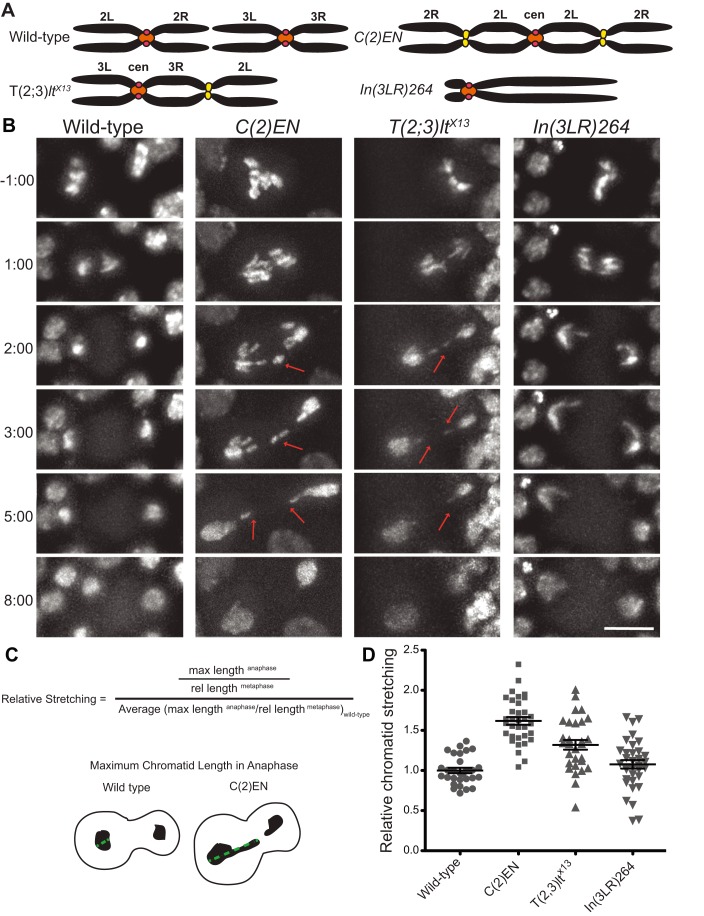
Displaced heterochromatin causes chromatin stretching during anaphase. (A) Schematic of three chromosome rearrangements: Compound Chromosome 2 (C(2)EN), Translocation (2;3)lt^X13^ (T(2;3)lt^X13^), and Inversion (3LR)264 (In(3LR)264). The first two rearrangements but not the third result in displaced heterochromatin. Pericentric heterochromatin surrounding the centromeres (red) is depicted in orange whereas displaced heterochromatin is denoted in yellow; (B) Images from live analysis of segregating anaphase chromosomes in each of the three rearrangements. C(2)EN and T(2;3)lt^X13^ show lagging chromatids that considerably stretch during anaphase (arrows), whereas In(3LR)264 shows a long chromatid with no stretching. Time 0∶00 equals anaphase onset and scale bars are 10 µm; (C) Schematics of relative stretching measurements: the longest anaphase chromatid length was measured as depicted in the bottom panel and normalized to its metaphase size and to the average control length; (D) Relative chromatid stretching in wild-type, C(2)EN, T(2;3)lt^X13^, and In(3LR)264 strains. Note that the average anaphase chromosome length of In (3LR) 264 is as predicted by its metaphase length, whereas C(2)EN and T(2;3)lt^X13^ exhibit longer chromatids; datasets can be found in Table S2.

The results above suggest that ectopic localization of cohesin can have an impact on its efficient removal from chromosomes and therefore may compromise chromosome disjunction during anaphase. Previous studies from analysis of fixed samples had already demonstrated that defects occur during segregation of compound chromosomes [31],[37], and recent studies demonstrate significant variation in arm length of the compound chromosomes during anaphase [38]. Thus, we decided to evaluate more carefully the segregation of these engineered chromosomes to determine the consequences of mislocalizing heterochromatin (and consequently cohesin enrichment). To assess the dynamics of chromosome segregation in these strains, we performed detailed live imaging of *Drosophila* neuroblasts carrying chromosomes with ectopic heterochromatin sites (C(2)EN and T(2;3)*lt^X13^*) (Figure 5A and 5B; Movies S6 and S7). Our analysis revealed that during anaphase, chromatids considerably stretch while segregating to opposite poles of the cell (Figure 5B–5D). We defined “stretched chromatin” as any detectable increase in length within a single chromatid whether it forms a detectable connection between sister chromatids (chromatin bridges) or not (most stretching did not resemble chromatin bridges).

To quantify anaphase chromosome stretching in the C (2) EN-bearing strains we have measured the length of the longest segregating chromatid from centromere to telomere and normalized it to the average anaphase chromatid length measured in wild-type cells (Figure 5C). As C(2)EN chromosomes are longer then wild-type chromosomes, all values were corrected for the expected length increase based on their metaphase size (see Figure S6 for metaphase length measurements). In most dividing cells, significant stretches of chromatin could be detected reaching longest chromatid lengths that are on average 62%±4% longer than the expected chromatid size (Figure 5D). To exclude that this observation was somehow related specifically to displaced Y-heterochromatin, we have analysed chromosome segregation in neuroblasts carrying the T(2;3)*lt^x13^* chromosome (this line contains pericentromeric heterochromatin and cohesin distal to the centromere; Figures 5A and S2). A significant amount of stretching was also observed in these chromosomes during anaphase displaying chromatids that are on average 32%±6% longer than their predicted size (Figure 5D). Importantly, the C(2)EN and T(2;3)*lt^x13^* observed stretching is specific to the presence of ectopic heterochromatin sites and not a consequence of increased chromosome size. Another engineered chromosome resulting in similarly long chromosomes but lacking significant amounts of mislocalized heterochromatic regions (ln (3LR) 264; Figure 5A), does not display significant stretching (Figure 5B and 5D; Movie S8).

**Figure 6:**
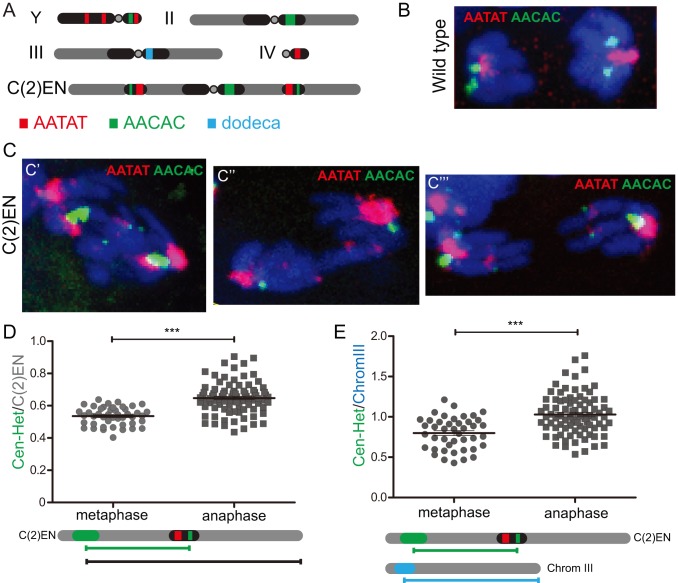
Relative position of ectopic heterochromatin domains reveals an anaphase-specific stretch. (A) Schematic representation of the chromosomal location of the probes used; (B) FISH of wild-type cells showing that in anaphase cells these probes detect only centromere-proximal regions; (C) FISH of anaphases in C(2)EN-bearing strains showing the position of ectopic heterochromatin regions labelled by the indicated probes. Note that in these cells the ectopic heterochromatin appears totally unresolved (C′), forming chromatin bridges (C′′) or already resolved (C′′′). Graphs show the relative position of ectopic heterochromatin regions of C(2)EN chromosomes relative (D) to its entire chromosome length and (E) to the length of Chromosome III; (*n*≥20, ***p<0.0001, two-tailed students *t*-test; datasets can be found in Table S2).

To confirm that the observed stretching is indeed caused by the ectopic heterochromatin we performed FISH analysis in fixed cells using heterochromatin-specific probes (Figure 6). We found that in all anaphases displaying lagging chromatin, this corresponded specifically to the C(2)EN chromosomes. These chromosomes often show unresolved chromatids containing the ectopic regions at the unresolved DNA (Figure 6C′). Importantly, even in the cells that display resolution of ectopic heterochromatin sites, we detect a significant lagging of these regions both in cells exhibiting chromatin bridges (Figure 6C′′) or displaying totally resolved chromosomes (Figure 6C′′′). If stretching is indeed caused by a “resistance” force placed specifically at the sites of mislocalized heterochromatin, we should expect that the stretching is more pronounced between the centromere and the ectopic region, while distal parts of the chromosome will be less prone to stretching. To test this, we have measured the relative position of the ectopic heterochromatin region and found that while in metaphase chromosomes the ectopic region localizes approximately in the middle of the chromatid, during anaphase this region is significantly lagging behind relative to its entire chromosome length (Figure 6E) and relatively to other chromosomes (Figure 6F). We therefore conclude that the ectopic heterochromatin present in C(2)EN strains induces anaphase-specific chromatin stretching between the centromere and the ectopic site.

To access whether this stretching behaviour is a general feature of chromosomes containing distal heterochromatic regions, we tested several inversions and translocations. All rearrangements in which pericentric heterochromatin is no longer adjacent to the centromere display ectopic constriction (Figure S7) and demonstrated some form of stretching behaviour, specific to the rearranged chromosome (Figure S8). In contrast, rearrangements where pericentric heterochromatin position is not affected rarely stretch (Figures S7, S8, and unpublished data). Importantly, the degree of stretching was greater in chromosomes that display a higher frequency of constrictions (and likely increased cohesin-mediated cohesion) and when the heterochromatin is placed more distal relative to the centromere (Figure S9).

**Figure 7:**
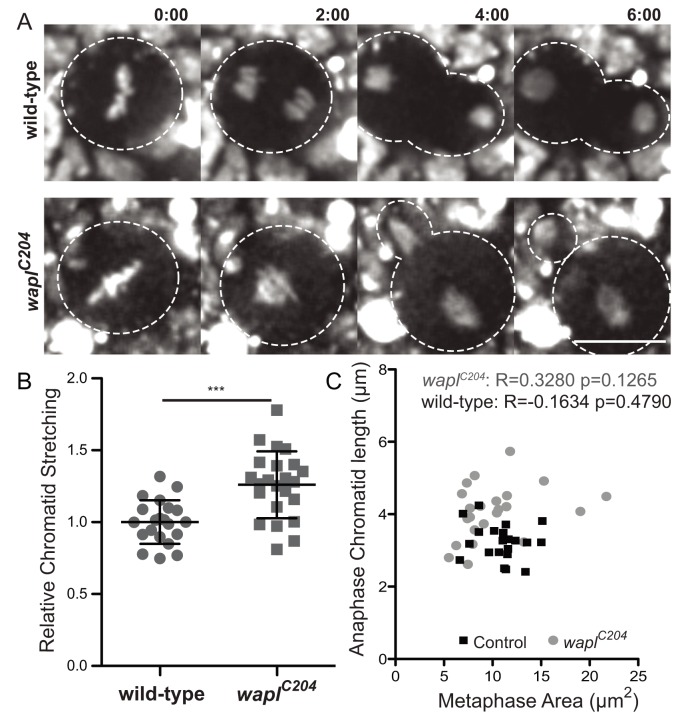
Wapl mutants exhibit chromosome stretching during anaphase. (A) Live-imaging of chromosomes in wild-type and *wapl^C204^* neuroblast cells. Time 0∶00 equals anaphase onset and scale bars is 10 µm. (B) Quantification of anaphase chromosome length in wild-type and *wapl^C204^*. *wapl^C204^* are significantly longer than wild-type cells (*n*≥10, ***p<0.0001, one-tailed students *t*-test; datasets can be found in Table S2); (C) Anaphase chromatid length plotted relatively to the metaphase area showing no significant correlation between stretched chromosomes and the organization of the metaphase plate.

The results above suggest that high levels of cohesin at sites distal to the centromere induce chromatin stretching during anaphase. To further test if there is a causal link between ectopic cohesion and chromosome stretching during anaphase, independent of the presence of heterochromatin, we evaluated the mitotic behaviour of *wapl* mutants. We measured the longest chromatid length during anaphase as above and found that in *wapl* mutants, chromosomes stretch considerably displaying an average length 26% longer than controls (Figure 7; Movie S9). We cannot rule out that defects in chromosome structure in *wapl^C204^* mutants may account for some of the observed stretching. Wapl mutations have a mild effect on sister chromatid cohesion ([34] and unpublished data). To control for this we have measured the area occupied by chromosomes during metaphase, as cohesion defects should lead to a more scattered metaphase figure. Although a few cells (∼8%) in *wapl^C204^* mutants display more scattered chromosomes in metaphase, the anaphase stretch does not correlate with the morphology of metaphase figures (Figure 7C) implying that the detected stretching occurs mostly during anaphase. We therefore conclude that high levels of cohesin along chromosome arms, whether induced by misplaced heterochromatin or impairment of cohesin removal from chromosome arms, is sufficient to compromise the efficiency of chromosome segregation and induce chromatin stretching during the late stages of mitosis.

## Discussion

Proper regulation of cohesin loading and release is essential for normal mitosis. Here we demonstrate that chromosome rearrangements can have profound influence on cohesin distribution and dynamics resulting in disruptions in chromosome segregation. Specifically, we find that heterochromatin is sufficient to induce accumulation of cohesin independent of centromere proximity. This accumulation leads to cohesin-dependent constriction of the ectopic heterochromatin at metaphase followed by abnormal chromosome stretching during anaphase.

In contrast to budding yeast, where cohesin accumulation is centromere-driven [17]–[20], our results suggest that in *Drosophila*, and likely other metazoa, the accumulation of cohesin complexes is mediated by heterochromatin. The factors that promote cohesin enrichment at heterochromatin remain unclear, but it is unlikely that H3diMeK9/HP1 are alone sufficient to induce cohesin's recruitment. Although cohesin and H3diMeK9 colocalize in most chromosomes, this correlation is not observed in all chromosomes, as the small Chromosome IV and the ectopic heterochromatin in T(2,3)*lt^X13^* contain high levels of cohesin despite having very little diMeK9 (Figure 1B and unpublished data). Additionally, tethering HP1 to sites distal to the centromere does not induce ectopic pairing or cohesion [39]. Therefore, heterochromatin-dependent loading of cohesin likely depends on several heterochromatic markers.

Accumulation of cohesin at pericentromeric regions during metaphase has been largely attributed to the protection of centromere-proximal complexes from the releasing pathways that remove cohesin from chromosomes [10]–[15]. Several lines of evidence from this study support that cohesin accumulation at heterochromatic sites in Drosophila is due to preferential loading in addition to (or instead of) selective protection. First, the cohesin loading factor Nipped-B, is localized preferentially at heterochromatic regions during S-phase. Secondly, in *wapl^C204^* mutants, in which cohesin removal along chromosome arms is disrupted, preferential accumulation of cohesin at the pericentric heterochromatin is not perturbed. Finally, MEI-S332/Sgo1, involved in recruiting protein phosphatase 2A (PP2A) to the centromere and thereby prevent cohesin removal, is not localized at the ectopic heterochromatin sites. *Drosophila* may rely on alternative MEI-S332/Sgo1-independent pathways to recruit PP2A to the heterochromatin or possess a yet unidentified ortholog for Sgo1 other than Mei-S332. Indeed, *mei-S332* mutants do not show any evident sister chromatid cohesion defects in unperturbed mitosis and mild levels of SCS are only evident in cells arrested in metaphase for long periods of time and subjected to hypotonic treatment [40]. Alternatively, despite the existence of a bona-fide prophase pathway, as evidenced by the high levels of Rad21-EGFP at chromosome arms in *wapl* mutants [16], it is possible that this activity is not enough to compromise the even higher density of cohesin present at heterochromatic sites.

Our observation that chromosomes containing long heterochromatic regions distal to the centromere undergo significant stretching during anaphase strongly suggests that sister chromatid disjunction is compromised in these regions. In addition, when arm removal of cohesin is impaired as in *wapl^C204^* mutants, we could also detect a significant amount of stretching, implying that high levels of cohesin at chromosome arms are sufficient to compromise timely chromosome segregation. It should be noted that the stretching in Wapl mutants is not as severe as in some of the analysed translocations suggesting that either heterochromatin is particularly more prone to stretching (possibly due to increased levels of catenation) or that other functions of Wapl may counteract the stretching behaviour (e.g., chromatin rigidity). Nevertheless, in both conditions the kinetics of cohesin's removal is slightly delayed. Assuming that separase cleavage capacity is not rate limiting in these cells, our findings suggest that the activity of this enzyme and/or complete cohesin removal is less efficient at centromere-distal regions (chromosome arms), as previously suggested in budding yeast [41]. The detected delay, however, is relatively modest (all cohesin cleavage is completed within a few minutes). Chromatin stretching, in contrast, was observed even in late anaphase cells, when all cohesin should be degraded. It is therefore likely that chromatin stretching results, additionally, from impairment in resolving other chromatin linkages. The high accumulation of cohesin at ectopic heterochromatic regions prior to anaphase entry may alone act as an obstacle for timely resolution of sister chromatid intertwining. Previous studies have pointed out that cohesin removal is a prerequisite for efficient decatenation. In budding yeast, persistence of catenation after S-phase in minichromosomes was shown to depend on cohesin [42]. Studies with purified mammalian chromosomes further suggest that centromeric DNA decatenation depends on cohesin removal [43]. Our previous experiments have also demonstrated that if TopoII inhibitors are added to metaphase-arrested cells, then the segregation movement induced by artificial cleavage of cohesin is slowed down, implying that few catenenes are resolved during anaphase [7]. It should be emphasized, however, that in wild-type chromosomes, this reduced level of catenation does not lead to chromatin stretching during anaphase.

The question is then why are DNA linkages at the centromere vicinity more effectively removed, despite the high levels of cohesin? A possible explanation for the difference between pericentric and ectopic heterochromatin is that the spindle forces alone might provide additional force that favours both the removal of cohesin and the decatenation of DNA. In agreement with this hypothesis, we found that the degree of stretching correlates with the distance of the heterochromatin segment to the centromere (Figure S9), suggesting that more distal regions, subjected to lower spindle forces, are more difficult to resolve. Several studies support that resolution of decatenation is indeed favoured by spindle forces [42]–[45]. As such, high levels of cohesin in regions subjected to reduced spindle forces, are likely to enter anaphase with abnormally high levels of sister chromatid intertwining, leading to detectable chromatin stretching. If so, there is an important functional implication of placing pericentromeric heterochromatin (and thereby cohesion) around the site of spindle attachment (the centromere).

Our findings provide a possible mechanism by which chromosomal translocations involving long stretches of heterochromatin may induce mitotic errors, as the abnormal chromatin stretching could potentially be problematic to the cell. While our previous study did not detect any DNA damage in C(2)EN [38], it is possible that cells with multiple rearrangements have higher incidences of broken chromosomes. Furthermore, our previous work demonstrated that cells dividing long chromosomes elongate, a compensatory mechanism that prevents cutting long stretched chromosomes, thereby avoiding DNA damage and aneuploidy [38]. Importantly, other cell types where these compensatory mechanisms are less active may be more sensitive to mitotic errors. For example, *Drosophila* syncytial embryos of C(2)EN strains have an increased incidence of thick chromatin bridges and abortive nuclear division [37]. In addition to compensatory mechanisms, cells with detrimental consequences due to stretching may be selectively eliminated from the population, which could explain the maintenance of euploidy in these strains. Indeed, in many of these strains, we often observe abnormal reshaping of the membrane during mitosis and at times, furrow regression (unpublished data), suggesting possible cell death. These studies have widespread implications given that many cancers contain inversions and translocations. In fact, translocations involving constitutive heterochromatin derived from human Chromosome 1 are commonly found in haematopoietic and solid tumours, and display aberrant heterochromatin foci [46],[47]. This association may be due to the effects of heterochromatin on gene expression in neighbouring euchromatic regions [46]. However, it has also been shown that rearrangements in cancers associated with pericentromeric heterochromatin are highly complex suggesting a general genomic instability in this region [48]. It would be interesting to determine if these translocations also display segregation delays and abnormal chromosome stretching during anaphase. If so, this would reveal a previously undescribed mechanism leading to transformation and cancer development.

## Materials and Methods

### Fly Strains

C(2)EN [30], T(2;3)lt^X13^ [33] (kindly provided by Barbara Wakamoto) and In(3LR)264 (Bloomington number 1222) were previously described. Transgenic flies expressing Rad21-EGFP were produced as described below. For all the experiments described here, the line Rad21^wt^-EGFP2 was used after recombination with Rad21^ex15^ alleles [49] (Rec 2.21), with the exception of the analysis of cohesin degradation in the Wapl mutant background, for which the line Rad21^TEV^-EGFP3 recombined with Rad21^ex15^ was used [16], using *wapl^C204^* mutant flies kindly provided by Maurizio Gatti's lab [34]. For experiments involving × chromosome-linked *wapl^C204^* mutants, only male flies (harbouring only one × chromosome) were used. C(2)EN strains carrying various transgenes were derived as previously described [32]. Strains expressing Nipped-B-EGFP have been previously published [36]. Fly strains also expressed H2Av-mRFP1 to monitor DNA or CID-mRFP1 to monitor centromeres [50]. For RNAi experiments, elaV-Gal4 flies [51] were crossed to the appropriate Transgenic RNAi Project (TRiP, Harvard Medical School) stocks to drive expression of RNAi in the third instar larval brain (see Figure S10 for crossing schemes). TRiP strains used were Nipped-B (32406) and SA (33395). A table with all the strains used can be found in the supporting materials (Table S1).

### Production of Transgenic Flies Expressing Rad21-EGFP

To construct Rad21-EGFP expressing flies, we have produced EGFP-tagged constructs similar to the previously described pCaSpeR-tubpr-Rad21-myc_10_ vector [49]. First, the ORF of Rad21 was amplified using primers CTGAATTCAGCCACCATGGCTTTCTATGAGCACATTATTTTGG and ATGCTAGCGCGAACAATTTTTGGGTTTTCGAACG to clone Rad21 or Rad21^TEV^ (with TEV-cleavable sites) in pRNA-EGFP [52] using the EcoR1/Nhe1 sites, giving rise to the pRNA-Rad21-EGFP/pRNA-Rad21^TEV^-EGFP vectors. A fragment containing the C-terminal half of Rad21 (with and without TEV sites) and the EGFP tag (SwaI/SpeI) was excised from the pRNA-Rad21-EGFP/pRNA-Rad21^TEV^-EGFP and cloned in pCaSpeR-tubpr-Rad21-myc_10_ after excision of a corresponding myc tagged fragment (excised with SwaI/NheI; the NheI site was introduced in pCaSpeR-tubpr-Rad21-myc_10_, immediately after the stop codon, by site directed mutagenesis). The resulting pCaSpeR-tubpr-Rad21^wt^-EGFP and pCaSpeR-tubpr-Rad21^(550-3TEV)^-EGFP vectors were used for p-element mediated transformation carried out at BestGene. Several lines containing Rad21^wt^-EGFP or Rad21^TEV^-EGFP insertions on the 2nd and 3rd chromosome were established and shown to efficiently rescue the lethality associated with Rad21 null mutations.

### Brain Spreads and Immunofluorescence

DAPI spreads were performed by dissecting brains in 0.7% NaCl, which were subsequently incubated in 0.02 mM colchicine in 0.7% NaCl for 40 minutes (except for anaphase analysis in Figure S8) and hypotonic shocked in 0.5% sodium citrate for 4–5 minutes. Cells were then fixed in 1.85% formaldehyde:45% glacial acetic acid while squashing with a vice and then, directly transferred to liquid nitrogen. Slides were allowed to air dry before mounting in Dapi + Vectashield mounting medium. For immunofluorescence, brains were dissected in 0.7% NaCl, incubated with 100 µM colchicine for one hour, hypotonic shocked in 0.5% sodium citrate for 2–3 minutes, and fixed on a 5 µl drop of fixative (3.7% formaldehyde, 0.1% Triton-X100 in PBS) placed on top of a siliconized coverslip. After 30 seconds, the brains were squashed between the coverslip and a slide, allowed to fix for an additional 1 min and then placed on liquid nitrogen. Slides were further extracted with 0.1% Triton-X100 in PBS for 10 min, and proceeded for immunofluorescence following standard protocols. Primary antibodies were rat anti-CID (gift from Claudio E. Sunkel) used at 1∶5,000, guinea pig anti-Mei-S332 [53] used at 1∶5,000, and rabbit anti-H3K9Me2 (Upstate) used at 1∶200. Secondary antibodies conjugated with fluorescent dyes from Alexa series (Invitrogen) were used according to the manufacturer's instructions. To detect Rad21-EGFP we used a GFP-booster (Chromotek) at 1∶200.

### Fluorescence In Situ Hybridization

Brains from third instar larvae were dissected in PBS and transferred to 0.5% sodium citrate solution for 2 min. Brains were then fixed in 4% formaldehyde/PBS 0.1% Tween-20 for 40 min at room temperature. Fixed brains were washed sequentially in PBS, 2× SSCT buffer (0.3 M NaCl in 30 mM sodium citrate, 0.1% Tween-20), and 2× SSCT/50% formamide. Brains were then incubated in 2× SSCT/50% formamide at 92°C for 3 min in a thermo cycler to denaturate DNA. DNA probes were diluted in the hybridization buffer (20% dextran sulfate; 2× SSCT/50% formamide; 0.5 mg/ml salmon sperm DNA) and denatured 5 min at 92°C. The fluorescently labelled DNA probes were used as follows: 50 µM of Chr_Y A546-(AATAT)_6_, Chr_X (359 bp satellite DNA) A546-GGGATCGTTAGCACTGGTAATTAGCTGC, and Ch_3 (dodeca satellite DNA) Cy5-ACGGGACCAGTACGG DNA probes, and 5 µM of Chr_2 A488-(AACAC)_7_. Brains were incubated in the probe solution at 92°C, 5 min and left at 37°C overnight. After incubation with the probes, brains were washed in 2× SSCT buffer at 60°C for 10 min and again at room temperature for 5 min. Finally, brains were incubated in DAPI solution for at least 30 min (up to 2 hours) with gentle agitation and mounted in Vectashield mounting medium with DAPI (Vector Laboratories).

### Tissue Preparation

For images in Figures 2A, 3C, 3D, 5B, S3, S4B, and S5, third instar larval brains were dissected in PBS and slightly squashed between a slide and a coverslip by capillary forces as described previously [54]. The preparation was visualized immediately under the microscope and for a maximum period of 60 min. For images in Figures 2B, 3A, 4A, 4D, 7A, and S4A brains were dissected in Schneider medium supplemented with 10% FBS and intact brains were mounted on a glass-bottom petridish (MakTek), covered with an oxygen-permeable membrane (YSI membrane kit), and sealed with Voltalef oil 10S (VWR). This procedure allowed long-term imaging of brains for periods over 4 hours.

### Microscopy

Fixed samples (Figures 1B, 3B, S2, and S8 [spreads]) were observed with an inverted wide-field DeltaVision microscope (Applied Precision Inc.) equipped with a 100×1.4 oil immersion objective (Olympus) and a EMCCD camera (Roper Cascade II or Roper Cascade 1024). Images were deconvolved using SoftWoRx (Applied Precision Inc.). Images in Figures 1C, S6A, and S7 were acquired with a Zeiss Axioskop2 plus with AxioCam HRm CCD, Plan-NEOFLUAR 100X/1.3 NA Oil Objective using the Axiovision 4.6.3 software. For the images on Figures 2A, 3A, 3C, S3A, S4A, and S4B, time-lapse microscopy was performed with a spinning disk confocal microscope (Perkin Elmer) equipped with a 60× Silicon Immersion Oil objective (Olympus, NA 1.35) and a Hamamatsu C9100-13 EMCCD camera. For the time-lapse images on Figures 2B, 3D, 4A, 4D, 7, and S3B, a spinning disk confocal microscope (Andor Revolution XD system) equipped with a 60× Oil objective (Nikon, NA 1.4) and an Andor iXon 897 camera was used. FISH images (Figures 6, S1, and S8) were collected on the same microscope using a 100× Oil objective (Nikon, NA 1.4). Movies in Figures 5B and S6 were acquired with a Leica DMI6000B wide-field inverted microscope equipped with a Hamamatsu EM charge-coupled device (CCD) camera (ORCA C9100-02) with a binning of 1 and obtained using a 100× Plan-Apochromat objective with an NA of 1.4, followed by deconvolution with LeicaAF software using six iterations of a blind deconvolution algorithm with a 1.5 refractive index. All images were assembled using ImageJ software (http://rsb.info.nih.gov/ij/) and selected stills were processed with Photoshop.

### Measurements

Maximal anaphase chromatid length was determined by measuring the length of the longest chromatid, from the telomere to the centromere located at the tip of the chromosome mass at the pole. Measurements of the relative position of the ectopic heterochromatin where performed by measuring the distance between the indicated probes and the entire chromosome length measured in the DAPI channel. Projected 2D images (maximum intensity) were used for these measurements. ImageJ and Prism software (GraphPad) were used for image quantification and statistical analysis, respectively. An unpaired *t*-test with a 95% confidence was used to calculate the *p*-value for all statistical analysis. Final graphic representation was done using Prism software (GraphPad).

### Kinetics of Cohesin Degradation

One stack of 20 frames (20 µm) was acquired every 30 s to image a large region of a brain lobe for 2 hours using a 50% 488 laser with 400 ms exposure. Images were cropped to single neuroblasts and a 6 µm projection was used to quantify Rad21-EGFP levels. The same region of interest (comprising the Rad21-EGFP signal during mid-metaphase) was used throughout the time lapse to measure the mean fluorescence intensity from the time of nuclear envelope breakdown until telophase. After background subtraction, images were normalized to the value at mid-metaphase (3–4 minutes before anaphase onset). For each dataset, data points were fit to a sigmoid curve (Y = 1/[1+ exp(h(x− km))]). The slope of the curve (h) was used to compare the kinetics of cohesin cleavage in the different experimental conditions (Figure 5C). For each experimental condition, a minimum of ten neuroblasts from four different brains were analysed. For statistical analysis, an unpaired *t*-test (one-tailed) with a 95% confidence was used to calculate the *p*-value using Prism software (GraphPad).

## Supporting Information

Figure S1 **Ectopic heterochromatin regions in C(2)EN chromosomes can be labelled with probes against repetitive regions.** (A) Schematic representation of the chromosomal localization of the probes used; (B) Metaphase and interphase distribution of pericentromeric regions in wild-type cells; (C) Metaphase and interphase distribution of pericentromeric regions in C(2)EN bearing cells; Inset shows a higher magnification (1.5×) of the ectopic heterochromatin. These regions can be detected with AATAT (red) and AACAC (green) probes at chromosome arms. In interphase, the same genomic region is located as two distinct foci placed away from the centromeric cluster. DNA is shown in blue and scale bars are 2 µm.
(TIF)

Figure S2 **T(2;3)** ***lt^X13^*** **but not In(3LR)264 contains ectopic cohesin sites.** Immunofluorescence shows Rad21-EGFP (green) at the displaced heterochromatic site (arrow) that lack proximal centromeres (CID in blue) in T(2;3)*lt^X13^*. In(3LR)264 breakpoints occur in euchromatic regions, thus Rad21-EGFP solely localizes near CID at pericentromeric regions. DNA is shown in red and scale bars are 5 µm.
(TIF)

Figure S3 **Cohesin is loaded at higher levels at pericentric and ectopic heterochromatin.** (A) Left panel describes the quantification of the relative fluorescence intensity between heterochromatic regions (encircled by the red line) and euchromatic regions (encircled by the green line) in wild-type cells. Right panel shows the relative fluorescence intensity for both HisH2AvD-mRFP and Rad21-EGFP; datasets can be found in Table S2; (B) Images from live analysis of the wild-type (top) and C(2)EN (bottom) strains expressing Rad2-EGFP (green). Note that whereas in wild-type cells Rad21 is enriched solely at the pericentromeric cluster, in C(2)EN strains two additional foci are observed in chromatin rich regions (arrows). DNA is labelled with Hoechst (red).
(TIF)

Figure S4 **Cohesin (Rad21) and the cohesin loader (Nipped-B) localize near centromeres during S-phase.** (A) Live imaging of CID-mRFP1 and Rad21-EGFP in wild-type neuroblast cells demonstrates that cohesin is highly enriched near, but not directly at, centromeres. Times are relative to anaphase onset (t = 0) and scale bars are 5 µm. (B) Live imaging of CID-mRFP1 and Nipped-B-EGFP in wild-type neuroblast cells demonstrates that during S-phase, the cohesin loader localizes similarly as Rad21-EGFP near centromeres but it is absent during mitosis. Scale bar is 10 µm.
(TIF)

Figure S5 **Ectopic chromatin regions in C(2)EN bearing cells display Heterochromatin Protein 1 (HP1) as two distinct foci during interphase.** Stills from live-cell imaging of wild-type (top panels) and C(2)EN bearing cells (bottom panels), expressing HP1-EGFP (green) and HisH2AvD-mRFP1 (red) in Drosophila larval neuroblasts. Times 0∶00 equals anaphase onset. In wild-type cells HP1 is visible only at the pericentromeric cluster, whereas in C(2)EN bearing cells two additional foci at a distance from the centromeres, are observed soon after the previous mitosis.
(TIF)

Figure S6 **Relative chromatid length during anaphase.** (A) Schematic representation of measurement of the relative chromatid length measured in metaphase spreads; (B) Graph with the relative chromatid length measured in metaphase for all the inversions/translocations used in this study. Each rearranged chromatid was measured relative to the entire length of chromosome × (for wild-type cells, the chromatid of Chromosome 3 was used). Bars represent average ± standard error of the mean (SEM); datasets can be found in Table S2.
(TIF)

Figure S7 **Rearranged and engineered chromosomes display ectopic constrictions at displaced heterochromatin sites in metaphase.** Metaphase spreads from several inversions and translocations. Percentages indicate the frequency of observed ectopic constrictions. The schematic diagram displays the heterochromatin placement in each strain (in black). Vertical lines indicate inverted or translocated breakpoints.
(TIF)

Figure S8 **Chromosomes with displaced heterochromatin stretch in anaphase.** (A) Images of anaphase figures in brain spreads (left) or FISH stainings of intact brains (right). Schematics depict heterochromatin placement in inversions and translocations. Black horizontal lines indicate the length of the rearranged chromatid relative to the entire length of chromosome × (average ± standard error of the mean (SEM); length of 3rd chromosome in controls). Red horizontal lines indicate the distance from the centromere to the ectopic heterochromatin region. Vertical lines indicate inverted or translocated breakpoints; (B) Frequency of normal, stretched, or unresolved anaphase figures obtained from fixed anaphase spreads of the various strains containing inversions and translocations; datasets can be found in Table S2; (C) Box plot of the relative stretching of each rearranged chromosome in anaphase analysed by live cell imaging. Each value was first normalized by the relative chromosome length measured in metaphase and subsequently by the average length observed for wild-type cells (see equation on Figure 5C and datasets in Table S2).
(TIF)

Figure S9 **Correlation of anaphase chromatin stretching with the distance from the centromere (A) or the frequency of observed constriction (B).** Datasets can be found in Table S2.
(TIF)

Figure S10 **Schematic representation of the crossing strategy for RNAi experiments in the C(2)EN strain.**
(TIF)

Table S1 **List of** ***Drosophila*** **stocks used in this study.**
(PDF)

Table S2 **Datasets for** **Figures 1D** **,** **4B, 4C** **,** **5D** **,** **6D, 6E** **,** **7B, 7C**, **S3A, S6B, S8B, S8C, S9A, and S9B.**
(XLSX)

Movie S1 **Dividing wild-type neuroblast expressing Rad21-EGFP (green and right panel) and H2AvD-mRFP1 (red).**
(MOV)

Movie S2 **Neuroblasts expressing Rad21-EGFP in wild-type (left) and C(2)EN (right) strains.**
(MOV)

Movie S3 **Dividing neuroblast expressing Rad21-EGFP (green) and H2AvD-mRFP1 (red) in** ***wapl^C204^*** **mutant strains.**
(MOV)

Movie S4 **Dividing neuroblasts expressing NippedB-EGFP (green) and H2AvD-mRFP1 (red) in wild-type cells.**
(MOV)

Movie S5 **Dividing neuroblast in wild-type cells (DNA labelled with H2AvD-mRFP1).**
(MOV)

Movie S6 **Dividing neuroblast in strains carrying the C(2)EN chromosome (DNA labelled with H2AvD-mRFP1).**
(MOV)

Movie S7 **Dividing neuroblast in strains carrying the T(2;3)** ***lt^X13^*** **translocation (DNA labelled with H2AvD-mRFP1).**
(MOV)

Movie S8 **Dividing neuroblast in strains carrying the Inversion In(3LR)264 (DNA labelled with H2AvD-mRFP1).**
(MOV)

Movie S9 **Dividing neuroblasts in control (left) and** ***wapl^C204^*** **(right) strains. DNA labelled with H2AvD-mRFP1.**
(MOV)

## Abbreviations

FISH: fluorescent in situ hybridization
RNAi: RNA interference
SA: stromalin
SCS: sister chromatid separation

## References

[1] Topp CN, Dawe RK (2006) Reinterpreting pericentromeric heterochromatin. Curr Opin Plant Biol 9: 647–653. 17015032 10.1016/j.pbi.2006.09.008

[2] Guacci V, Koshland D, Strunnikov A (1997) A direct link between sister chromatid cohesion and chromosome condensation revealed through the analysis of MCD1 in S. cerevisiae. Cell 91: 47–57. 9335334 10.1016/s0092-8674(01)80008-8 PMC2670185

[3] Michaelis C, Ciosk R, Nasmyth K (1997) Cohesins: chromosomal proteins that prevent premature separation of sister chromatids. Cell 91: 35–45. 9335333 10.1016/s0092-8674(01)80007-6

[4] Haering CH, Farcas AM, Arumugam P, Metson J, Nasmyth K (2008) The cohesin ring concatenates sister DNA molecules. Nature 454: 297–301. 18596691 10.1038/nature07098

[5] Uhlmann F, Lottspeich F, Nasmyth K (1999) Sister-chromatid separation at anaphase onset is promoted by cleavage of the cohesin subunit Scc1. Nature 400: 37–42. 10403247 10.1038/21831

[6] Uhlmann F, Wernic D, Poupart MA, Koonin EV, Nasmyth K (2000) Cleavage of cohesin by the CD clan protease separin triggers anaphase in yeast. Cell 103: 375–386. 11081625 10.1016/s0092-8674(00)00130-6

[7] Oliveira RA, Hamilton RS, Pauli A, Davis I, Nasmyth K (2010) Cohesin cleavage and Cdk inhibition trigger formation of daughter nuclei. Nat Cell Biol 12: 185–192. 20081838 10.1038/ncb2018 PMC3284228

[8] Warren WD, Steffensen S, Lin E, Coelho P, Loupart M, et al (2000) The Drosophila RAD21 cohesin persists at the centromere region in mitosis. Curr Biol 10: 1463–1466. 11102811 10.1016/s0960-9822(00)00806-x

[9] Gerlich D, Koch B, Dupeux F, Peters JM, Ellenberg J (2006) Live-Cell Imaging Reveals a Stable Cohesin-Chromatin Interaction after but Not before DNA Replication. Curr Biol 16: 1571–1578. 16890534 10.1016/j.cub.2006.06.068

[10] McGuinness BE, Hirota T, Kudo NR, Peters JM, Nasmyth K (2005) Shugoshin prevents dissociation of cohesin from centromeres during mitosis in vertebrate cells. PLoS Biol 3: e86. 15737064 10.1371/journal.pbio.0030086 PMC1054882

[11] Waizenegger IC, Hauf S, Meinke A, Peters JM (2000) Two distinct pathways remove mammalian cohesin from chromosome arms in prophase and from centromeres in anaphase. Cell 103: 399–410. 11081627 10.1016/s0092-8674(00)00132-x

[12] Hauf S, Roitinger E, Koch B, Dittrich CM, Mechtler K, et al (2005) Dissociation of cohesin from chromosome arms and loss of arm cohesion during early mitosis depends on phosphorylation of SA2. PLoS Biol 3: e69. 15737063 10.1371/journal.pbio.0030069 PMC1054881

[13] Losada A, Hirano M, Hirano T (1998) Identification of Xenopus SMC protein complexes required for sister chromatid cohesion. Genes Dev 12: 1986–1997. 9649503 10.1101/gad.12.13.1986 PMC316973

[14] Gimenez-Abian JF, Sumara I, Hirota T, Hauf S, Gerlich D, et al (2004) Regulation of sister chromatid cohesion between chromosome arms. Curr Biol 14: 1187–1193. 15242616 10.1016/j.cub.2004.06.052

[15] Sumara I, Vorlaufer E, Gieffers C, Peters BH, Peters JM (2000) Characterization of vertebrate cohesin complexes and their regulation in prophase. J Cell Biol 151: 749–762. 11076961 10.1083/jcb.151.4.749 PMC2169443

[16] Eichinger CS, Kurze A, Oliveira RA, Nasmyth K (2013) Disengaging the Smc3/kleisin interface releases cohesin from Drosophila chromosomes during interphase and mitosis. EMBO J 32: 656–665. 23340528 10.1038/emboj.2012.346 PMC3590983

[17] Weber SA, Gerton JL, Polancic JE, DeRisi JL, Koshland D, et al (2004) The kinetochore is an enhancer of pericentric cohesin binding. PLoS Biol 2: E260. 15309047 10.1371/journal.pbio.0020260 PMC490027

[18] Tanaka T, Cosma MP, Wirth K, Nasmyth K (1999) Identification of cohesin association sites at centromeres and along chromosome arms. Cell 98: 847–858. 10499801 10.1016/s0092-8674(00)81518-4

[19] Lengronne A, Katou Y, Mori S, Yokobayashi S, Kelly GP, et al (2004) Cohesin relocation from sites of chromosomal loading to places of convergent transcription. Nature 430: 573–578. 15229615 10.1038/nature02742 PMC2610358

[20] Hu B, Itoh T, Mishra A, Katoh Y, Chan KL, et al (2010) ATP hydrolysis is required for relocating cohesin from sites occupied by its Scc2/4 loading complex. Curr Biol 21: 12–24. 21185190 10.1016/j.cub.2010.12.004 PMC4763544

[21] Nonaka N, Kitajima T, Yokobayashi S, Xiao G, Yamamoto M, et al (2002) Recruitment of cohesin to heterochromatic regions by Swi6/HP1 in fission yeast. Nat Cell Biol 4: 89–93. 11780129 10.1038/ncb739

[22] Bernard P, Maure JF, Partridge JF, Genier S, Javerzat JP, et al (2001) Requirement of heterochromatin for cohesion at centromeres. Science 294: 2539–2542. 11598266 10.1126/science.1064027

[23] Inoue A, Hyle J, Lechner MS, Lahti JM (2008) Perturbation of HP1 localization and chromatin binding ability causes defects in sister-chromatid cohesion. Mutat Res 657: 48–55. 18790078 10.1016/j.mrgentox.2008.08.010

[24] Shimura M, Toyoda Y, Iijima K, Kinomoto M, Tokunaga K, et al (2011) Epigenetic displacement of HP1 from heterochromatin by HIV-1 Vpr causes premature sister chromatid separation. J Cell Biol 194: 721–735. 21875947 10.1083/jcb.201010118 PMC3171121

[25] Hahn M, Dambacher S, Dulev S, Kuznetsova AY, Eck S, et al (2013) Suv4-20h2 mediates chromatin compaction and is important for cohesin recruitment to heterochromatin. Genes Dev 27: 859–872. 23599346 10.1101/gad.210377.112 PMC3650224

[26] Serrano A, Rodriguez-Corsino M, Losada A (2009) Heterochromatin protein 1 (HP1) proteins do not drive pericentromeric cohesin enrichment in human cells. PLoS ONE 4: e5118. 19352502 10.1371/journal.pone.0005118 PMC2662427

[27] Koch B, Kueng S, Ruckenbauer C, Wendt KS, Peters JM (2008) The Suv39h-HP1 histone methylation pathway is dispensable for enrichment and protection of cohesin at centromeres in mammalian cells. Chromosoma 117: 199–210. 18075750 10.1007/s00412-007-0139-z

[28] Gandhi R, Gillespie PJ, Hirano T (2006) Human Wapl is a cohesin-binding protein that promotes sister-chromatid resolution in mitotic prophase. Curr Biol 16: 2406–2417. 17112726 10.1016/j.cub.2006.10.061 PMC1850625

[29] Kueng S, Hegemann B, Peters BH, Lipp JJ, Schleiffer A, et al (2006) Wapl controls the dynamic association of cohesin with chromatin. Cell 127: 955–967. 17113138 10.1016/j.cell.2006.09.040

[30] Novitski E, Grace D, Strommen C (1981) The entire compound autosomes of Drosophila melanogaster. Genetics 98: 257–273. 6799353 10.1093/genetics/98.2.257 PMC1214438

[31] Gonzalez C, Casal Jimenez J, Ripoll P, Sunkel CE (1991) The spindle is required for the process of sister chromatid separation in Drosophila neuroblasts. Exp Cell Res 192: 10–15. 1898588 10.1016/0014-4827(91)90150-s

[32] Martins T, Kotadia S, Malmanche N, Sunkel CE, Sullivan W (2013) Strategies for outcrossing and genetic manipulation of Drosophila compound autosome stocks. G3 (Bethesda) 3: 1–4. 23316433 10.1534/g3.112.004481 PMC3538334

[33] Wakimoto BT, Hearn MG (1990) The effects of chromosome rearrangements on the expression of heterochromatic genes in chromosome 2L of Drosophila melanogaster. Genetics 125: 141–154. 2111264 10.1093/genetics/125.1.141 PMC1203996

[34] Verni F, Gandhi R, Goldberg ML, Gatti M (2000) Genetic and molecular analysis of wings apart-like (wapl), a gene controlling heterochromatin organization in Drosophila melanogaster. Genetics 154: 1693–1710. 10747063 10.1093/genetics/154.4.1693 PMC1461031

[35] Clarke A, Orr-Weaver TL (2006) Sister chromatid cohesion at the centromere: confrontation between kinases and phosphatases? Dev Cell 10: 544–547. 16678770 10.1016/j.devcel.2006.04.017

[36] Gause M, Misulovin Z, Bilyeu A, Dorsett D (2010) Dosage-sensitive regulation of cohesin chromosome binding and dynamics by Nipped-B, Pds5, and Wapl. Mol Cell Biol 30: 4940–4951. 20696838 10.1128/MCB.00642-10 PMC2950535

[37] Sullivan W, Daily DR, Fogarty P, Yook KJ, Pimpinelli S (1993) Delays in anaphase initiation occur in individual nuclei of the syncytial Drosophila embryo. Mol Biol Cell 4: 885–896. 8257792 10.1091/mbc.4.9.885 PMC275719

[38] Kotadia S, Montembault E, Sullivan W, Royou A (2012) Cell elongation is an adaptive response for clearing long chromatid arms from the cleavage plane. J Cell Biol 199: 745–753. 23185030 10.1083/jcb.201208041 PMC3514784

[39] Olszak AM, van Essen D, Pereira AJ, Diehl S, Manke T, et al (2011) Heterochromatin boundaries are hotspots for de novo kinetochore formation. Nat Cell Biol 13: 799–808. 21685892 10.1038/ncb2272

[40] LeBlanc HN, Tang TT, Wu JS, Orr-Weaver TL (1999) The mitotic centromeric protein MEI-S332 and its role in sister-chromatid cohesion. Chromosoma 108: 401–411. 10654079 10.1007/s004120050392

[41] Renshaw MJ, Ward JJ, Kanemaki M, Natsume K, Nedelec FJ, et al (2010) Condensins promote chromosome recoiling during early anaphase to complete sister chromatid separation. Dev Cell 19: 232–244. 20708586 10.1016/j.devcel.2010.07.013 PMC2938479

[42] Farcas AM, Uluocak P, Helmhart W, Nasmyth K (2011) Cohesin's concatenation of sister DNAs maintains their intertwining. Mol Cell 44: 97–107. 21981921 10.1016/j.molcel.2011.07.034 PMC3240746

[43] Wang LH, Mayer B, Stemmann O, Nigg EA (2010) Centromere DNA decatenation depends on cohesin removal and is required for mammalian cell division. J Cell Sci 123: 806–813. 20144989 10.1242/jcs.058255

[44] Kenney RD, Heald R (2006) Essential roles for cohesin in kinetochore and spindle function in Xenopus egg extracts. J Cell Sci 119: 5057–5066. 17158911 10.1242/jcs.03277

[45] Baxter J, Sen N, Martinez VL, De Carandini ME, Schvartzman JB, et al (2011) Positive supercoiling of mitotic DNA drives decatenation by topoisomerase II in eukaryotes. Science 331: 1328–1332. 21393545 10.1126/science.1201538

[46] Fournier A, McLeer-Florin A, Lefebvre C, Duley S, Barki L, et al (2010) 1q12 chromosome translocations form aberrant heterochromatic foci associated with changes in nuclear architecture and gene expression in B cell lymphoma. EMBO Mol Med 2: 159–171. 20432501 10.1002/emmm.201000067 PMC3377314

[47] Busson-Le Coniat M, Salomon-Nguyen F, Dastugue N, Maarek O, Lafage-Pochitaloff M, et al (1999) Fluorescence in situ hybridization analysis of chromosome 1 abnormalities in hematopoietic disorders: rearrangements of DNA satellite II and new recurrent translocations. Leukemia 13: 1975–1981. 10602418 10.1038/sj.leu.2401587

[48] Itoyama T, Nanjungud G, Chen W, Dyomin VG, Teruya-Feldstein J, et al (2002) Molecular cytogenetic analysis of genomic instability at the 1q12-22 chromosomal site in B-cell non-Hodgkin lymphoma. Genes Chromosomes Cancer 35: 318–328. 12378526 10.1002/gcc.10120

[49] Pauli A, Althoff F, Oliveira RA, Heidmann S, Schuldiner O, et al (2008) Cell-type-specific TEV protease cleavage reveals cohesin functions in Drosophila neurons. Dev Cell 14: 239–251. 18267092 10.1016/j.devcel.2007.12.009 PMC2258333

[50] Schuh M, Lehner CF, Heidmann S (2007) Incorporation of Drosophila CID/CENP-A and CENP-C into centromeres during early embryonic anaphase. Curr Biol 17: 237–243. 17222555 10.1016/j.cub.2006.11.051

[51] Lin DM, Goodman CS (1994) Ectopic and increased expression of Fasciclin II alters motoneuron growth cone guidance. Neuron 13: 507–523. 7917288 10.1016/0896-6273(94)90022-1

[52] McGuinness BE, Anger M, Kouznetsova A, Gil-Bernabe AM, Helmhart W, et al (2009) Regulation of APC/C activity in oocytes by a Bub1-dependent spindle assembly checkpoint. Curr Biol 19: 369–380. 19249208 10.1016/j.cub.2009.01.064

[53] Tang TT, Bickel SE, Young LM, Orr-Weaver TL (1998) Maintenance of sister-chromatid cohesion at the centromere by the Drosophila MEI-S332 protein. Genes Dev 12: 3843–3856. 9869638 10.1101/gad.12.24.3843 PMC317262

[54] Buffin E, Lefebvre C, Huang J, Gagou ME, Karess RE (2005) Recruitment of Mad2 to the kinetochore requires the Rod/Zw10 complex. Curr Biol 15: 856–861. 15886105 10.1016/j.cub.2005.03.052

