# Supplementary figures and images for: Centromere-Independent Accumulation of Cohesin at Ectopic Heterochromatin Sites Induces Chromosome Stretching during Anaphase
Source: PLoS Biol. 2014 Oct 7;12(10):e1001962. doi: 10.1371/journal.pbio.1001962 (PMC4188515; doi:10.1371/journal.pbio.1001962)

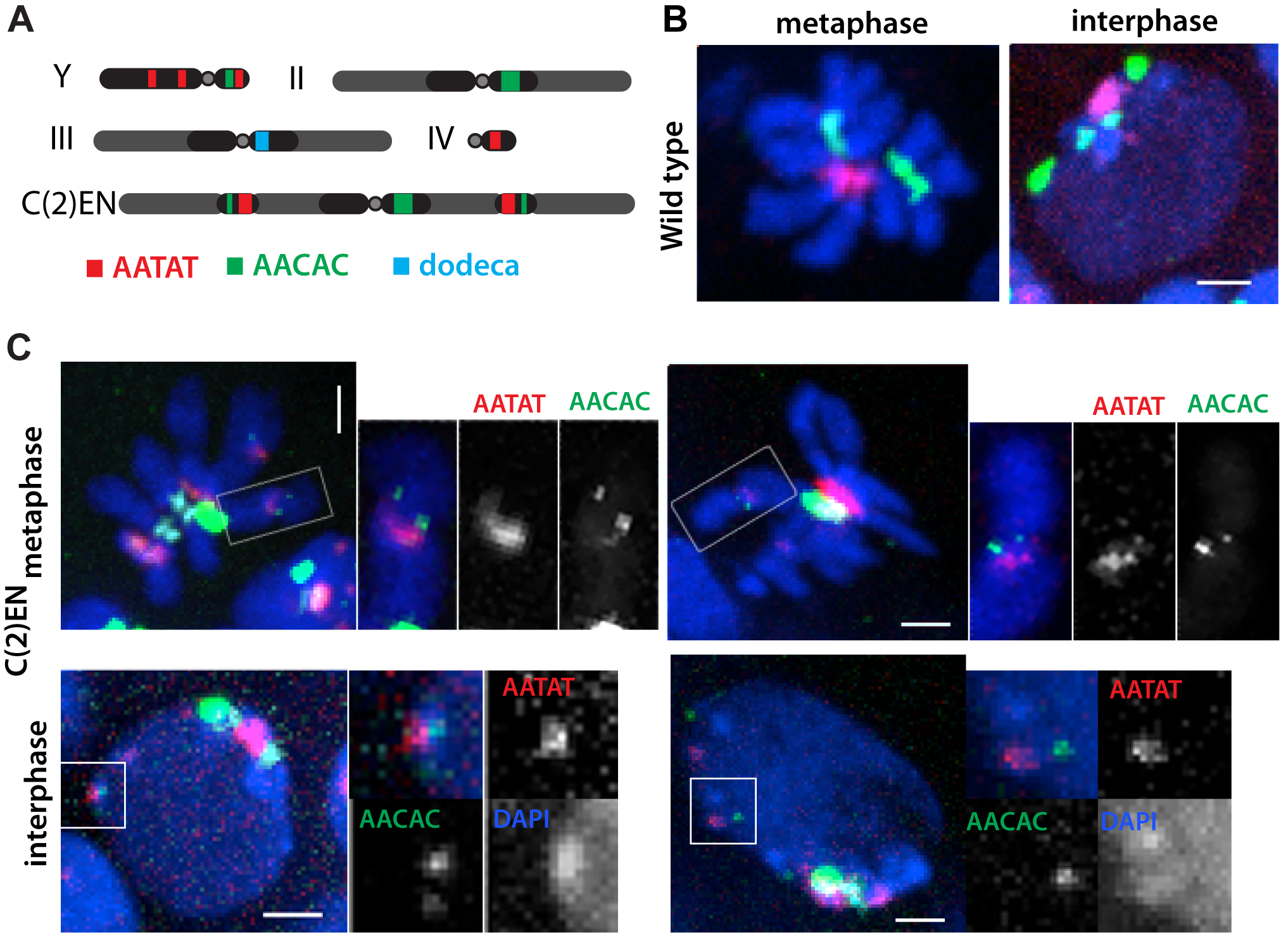

Supplement: Figure S1 — Ectopic heterochromatin regions in C(2)EN chromosomes can be labelled with probes against repetitive regions. (A) Schematic representation of the chromosomal localization of the probes used; (B) Metaphase and interphase distribution of pericentromeric regions in wild-type cells; (C) Metaphase and interphase distribution of pericentromeric regions in C(2)EN bearing cells; Inset shows a higher magnification (1.5×) of the ectopic heterochromatin. These regions can be detected with AATAT (red) and AACAC (green) probes at chromosome arms. In interphase, the same genomic region is located as two distinct foci placed away from the centromeric cluster. DNA is shown in blue and scale bars are 2 µm. (TIF) [file pbio.1001962.s001.tif]

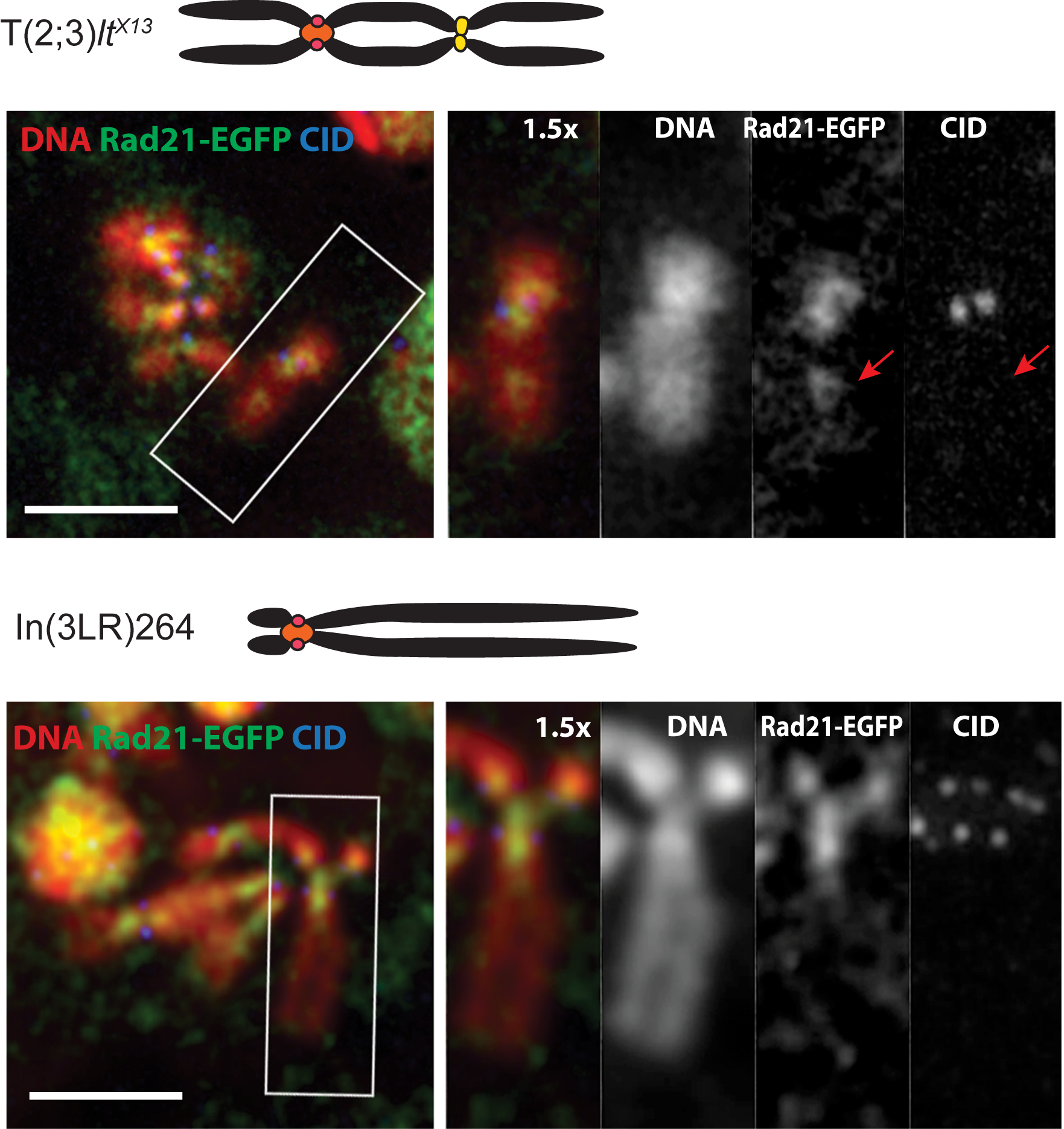

Supplement: Figure S2 — T(2;3) ltX13 but not In(3LR)264 contains ectopic cohesin sites. Immunofluorescence shows Rad21-EGFP (green) at the displaced heterochromatic site (arrow) that lack proximal centromeres (CID in blue) in T(2;3)ltX13. In(3LR)264 breakpoints occur in euchromatic regions, thus Rad21-EGFP solely localizes near CID at pericentromeric regions. DNA is shown in red and scale bars are 5 µm. (TIF) [file pbio.1001962.s002.tif]

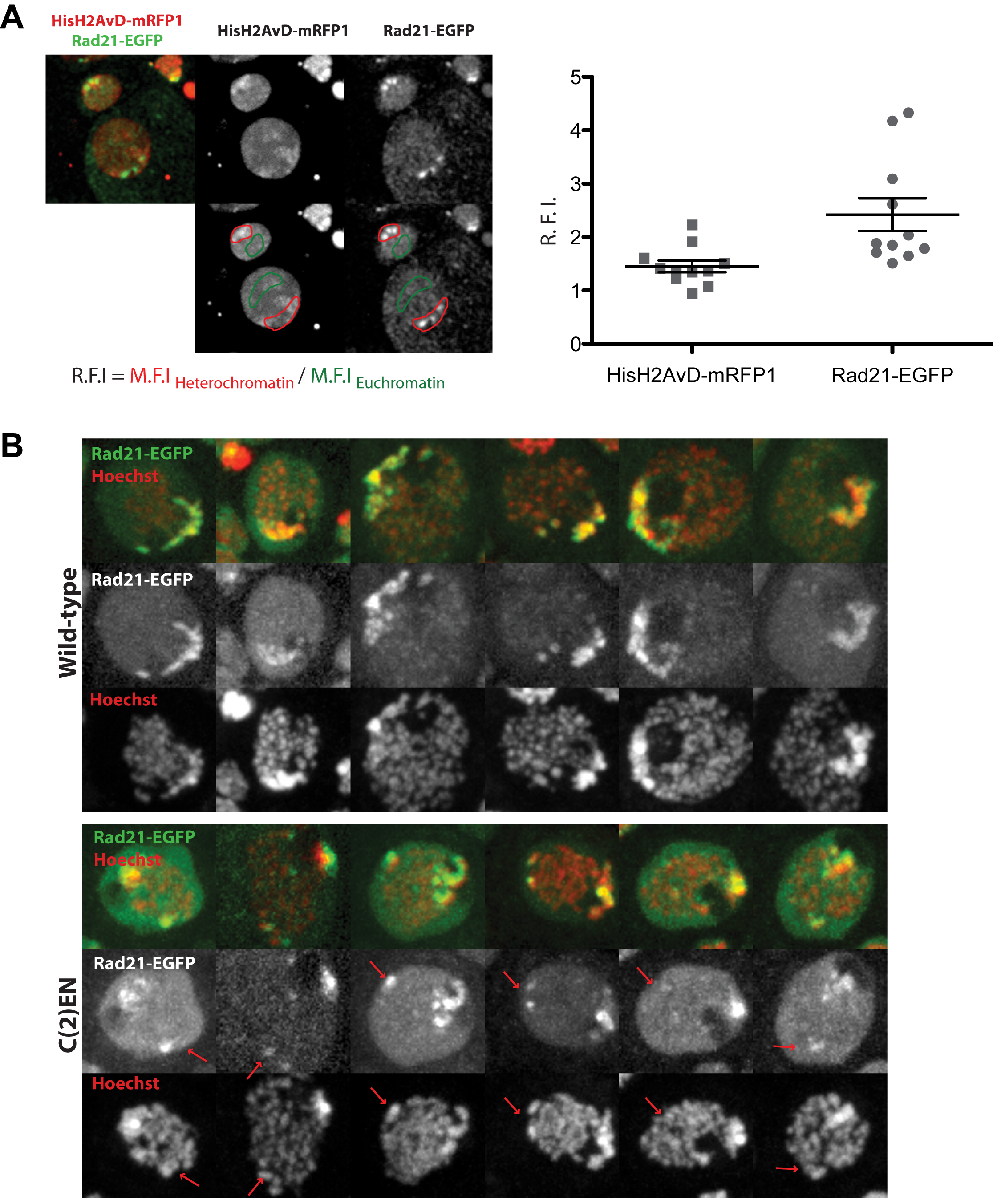

Supplement: Figure S3 — Cohesin is loaded at higher levels at pericentric and ectopic heterochromatin. (A) Left panel describes the quantification of the relative fluorescence intensity between heterochromatic regions (encircled by the red line) and euchromatic regions (encircled by the green line) in wild-type cells. Right panel shows the relative fluorescence intensity for both HisH2AvD-mRFP and Rad21-EGFP; datasets can be found in Table S2; (B) Images from live analysis of the wild-type (top) and C(2)EN (bottom) strains expressing Rad2-EGFP (green). Note that whereas in wild-type cells Rad21 is enriched solely at the pericentromeric cluster, in C(2)EN strains two additional foci are observed in chromatin rich regions (arrows). DNA is labelled with Hoechst (red). (TIF) [file pbio.1001962.s003.tif]

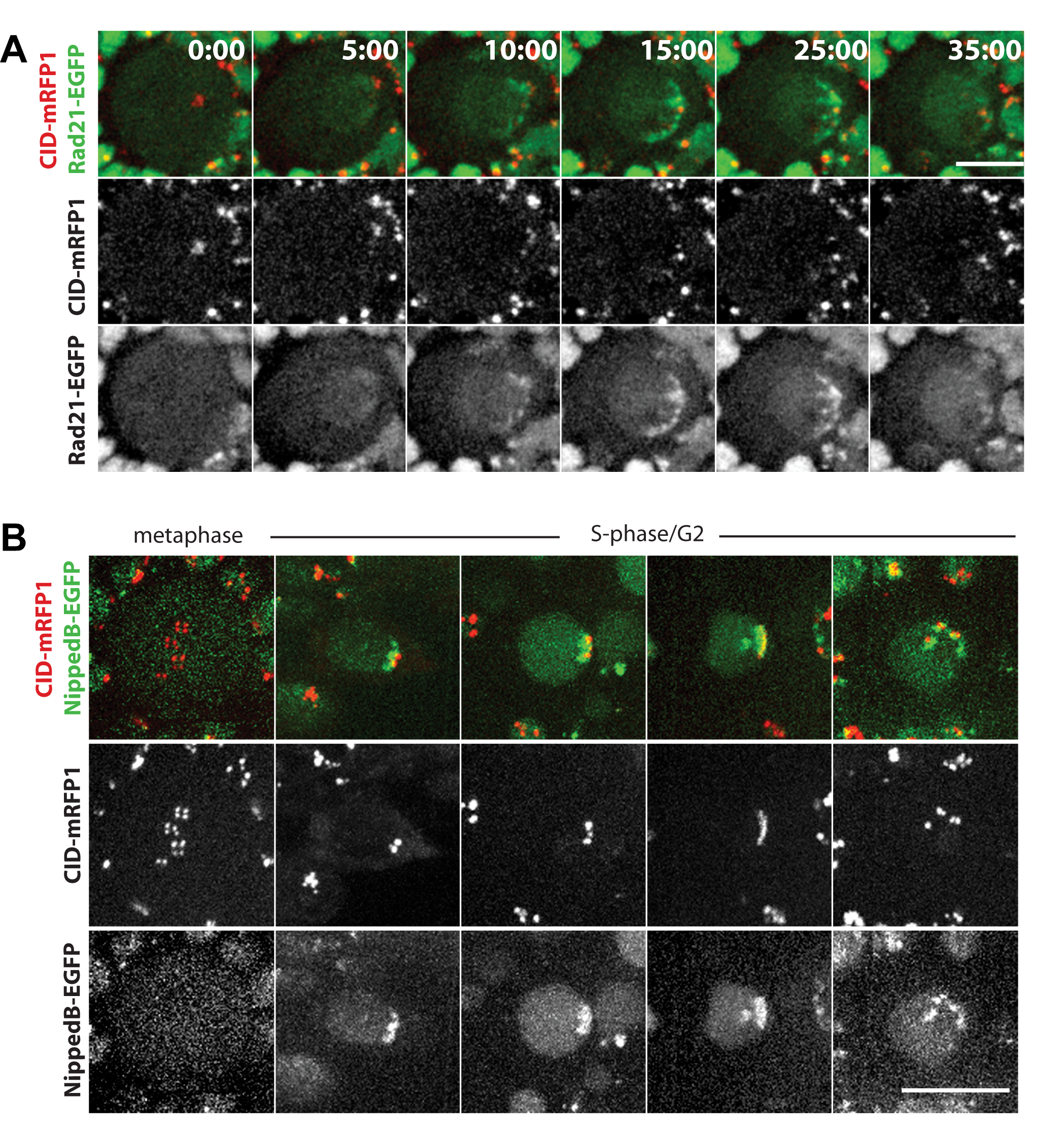

Supplement: Figure S4 — Cohesin (Rad21) and the cohesin loader (Nipped-B) localize near centromeres during S-phase. (A) Live imaging of CID-mRFP1 and Rad21-EGFP in wild-type neuroblast cells demonstrates that cohesin is highly enriched near, but not directly at, centromeres. Times are relative to anaphase onset (t = 0) and scale bars are 5 µm. (B) Live imaging of CID-mRFP1 and Nipped-B-EGFP in wild-type neuroblast cells demonstrates that during S-phase, the cohesin loader localizes similarly as Rad21-EGFP near centromeres but it is absent during mitosis. Scale bar is 10 µm. (TIF) [file pbio.1001962.s004.tif]

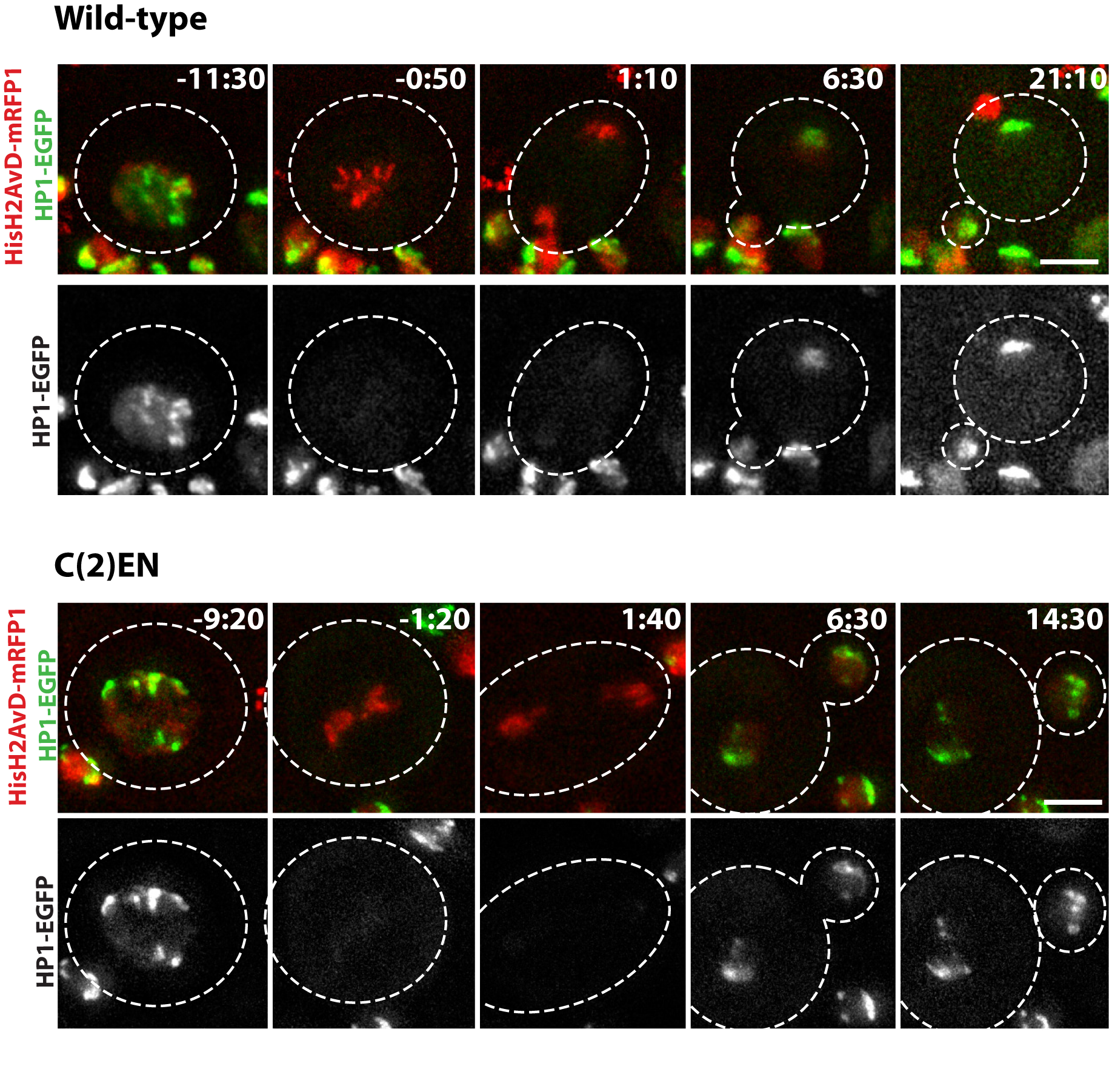

Supplement: Figure S5 — Ectopic chromatin regions in C(2)EN bearing cells display Heterochromatin Protein 1 (HP1) as two distinct foci during interphase. Stills from live-cell imaging of wild-type (top panels) and C(2)EN bearing cells (bottom panels), expressing HP1-EGFP (green) and HisH2AvD-mRFP1 (red) in Drosophila larval neuroblasts. Times 0∶00 equals anaphase onset. In wild-type cells HP1 is visible only at the pericentromeric cluster, whereas in C(2)EN bearing cells two additional foci at a distance from the centromeres, are observed soon after the previous mitosis. (TIF) [file pbio.1001962.s005.tif]

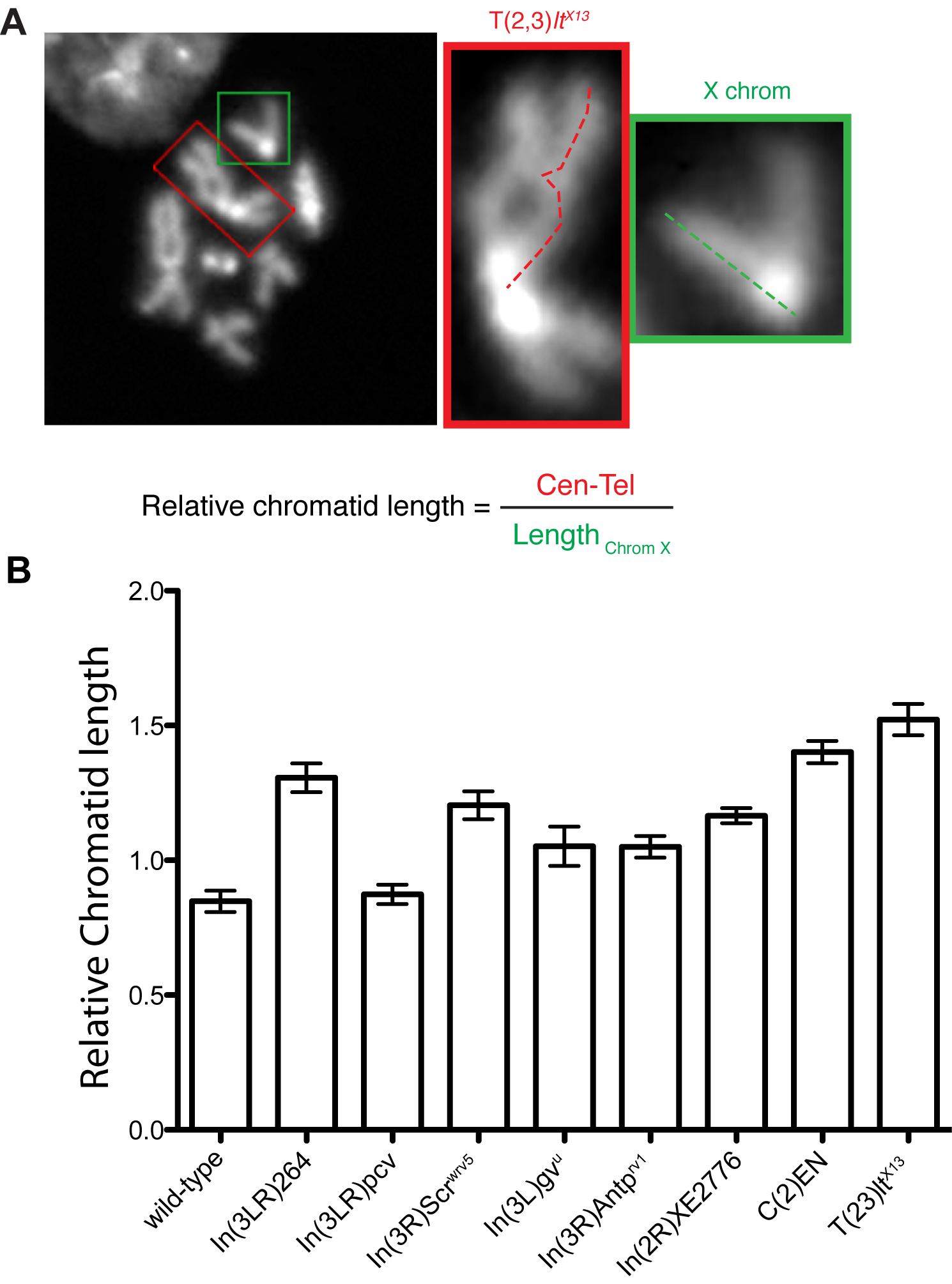

Supplement: Figure S6 — Relative chromatid length during anaphase. (A) Schematic representation of measurement of the relative chromatid length measured in metaphase spreads; (B) Graph with the relative chromatid length measured in metaphase for all the inversions/translocations used in this study. Each rearranged chromatid was measured relative to the entire length of chromosome × (for wild-type cells, the chromatid of Chromosome 3 was used). Bars represent average ± standard error of the mean (SEM); datasets can be found in Table S2. (TIF) [file pbio.1001962.s006.tif]

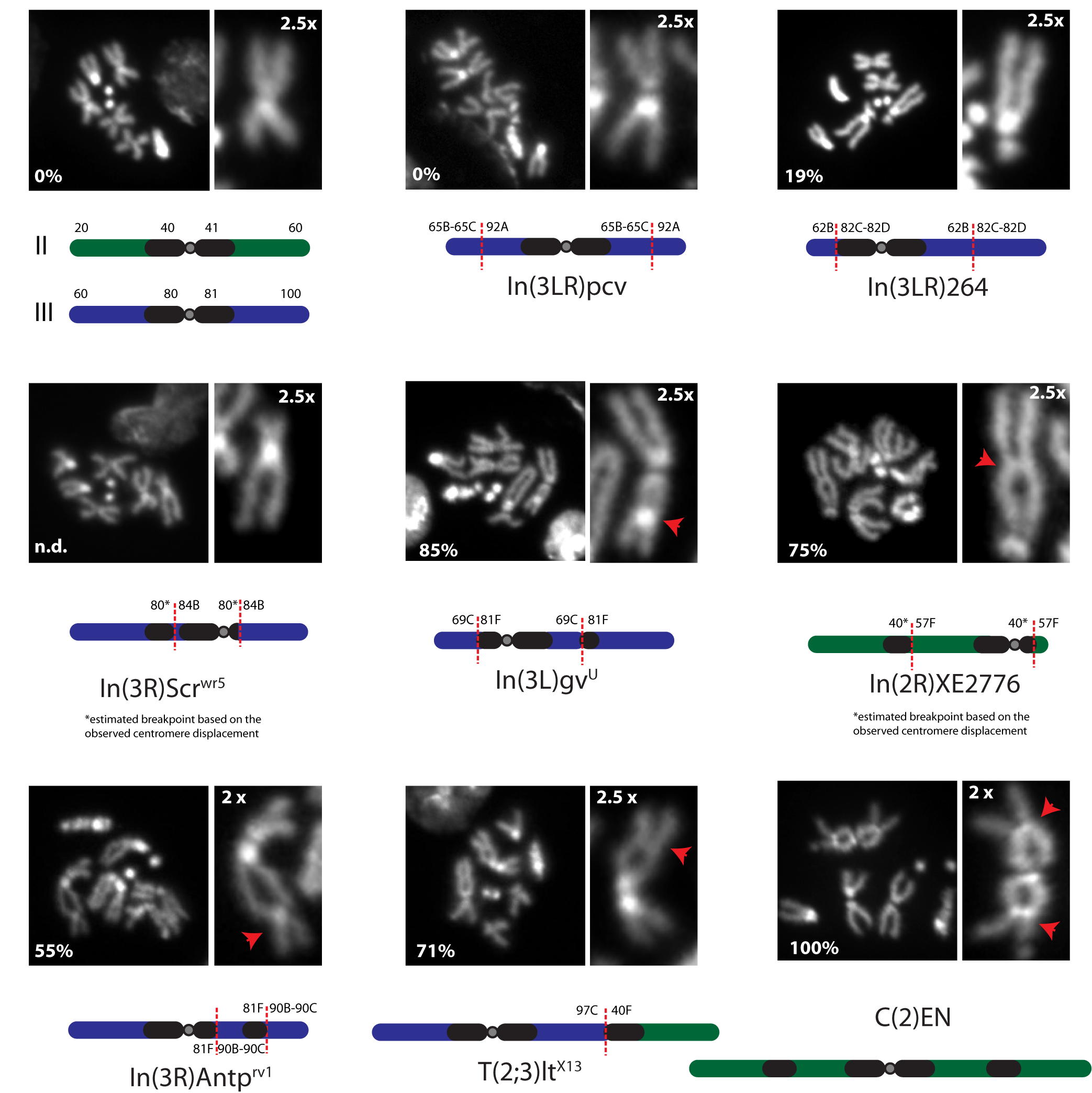

Supplement: Figure S7 — Rearranged and engineered chromosomes display ectopic constrictions at displaced heterochromatin sites in metaphase. Metaphase spreads from several inversions and translocations. Percentages indicate the frequency of observed ectopic constrictions. The schematic diagram displays the heterochromatin placement in each strain (in black). Vertical lines indicate inverted or translocated breakpoints. (TIF) [file pbio.1001962.s007.tif]

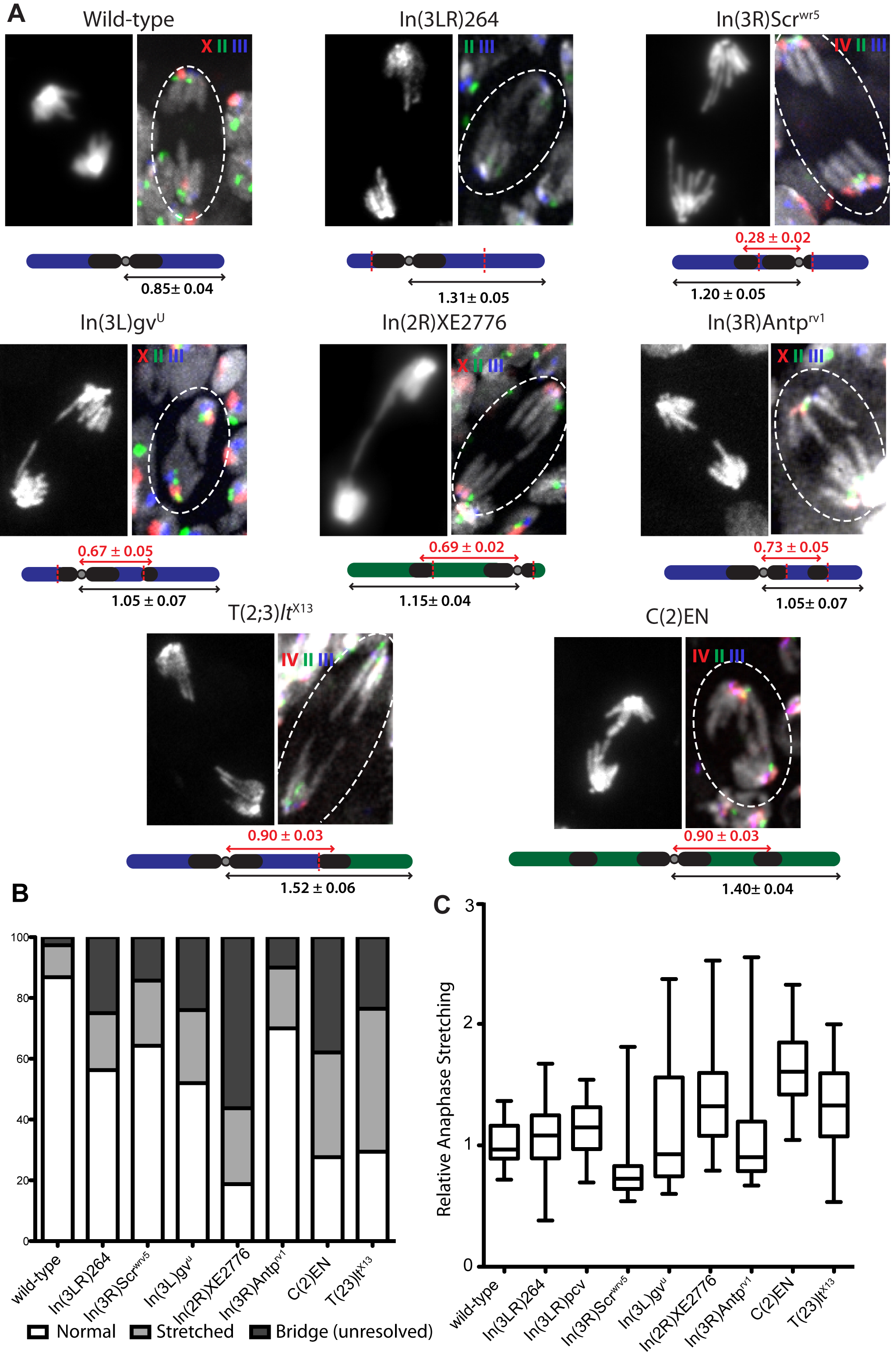

Supplement: Figure S8 — Chromosomes with displaced heterochromatin stretch in anaphase. (A) Images of anaphase figures in brain spreads (left) or FISH stainings of intact brains (right). Schematics depict heterochromatin placement in inversions and translocations. Black horizontal lines indicate the length of the rearranged chromatid relative to the entire length of chromosome × (average ± standard error of the mean (SEM); length of 3rd chromosome in controls). Red horizontal lines indicate the distance from the centromere to the ectopic heterochromatin region. Vertical lines indicate inverted or translocated breakpoints; (B) Frequency of normal, stretched, or unresolved anaphase figures obtained from fixed anaphase spreads of the various strains containing inversions and translocations; datasets can be found in Table S2; (C) Box plot of the relative stretching of each rearranged chromosome in anaphase analysed by live cell imaging. Each value was first normalized by the relative chromosome length measured in metaphase and subsequently by the average length observed for wild-type cells (see equation on Figure 5C and datasets in Table S2). (TIF) [file pbio.1001962.s008.tif]

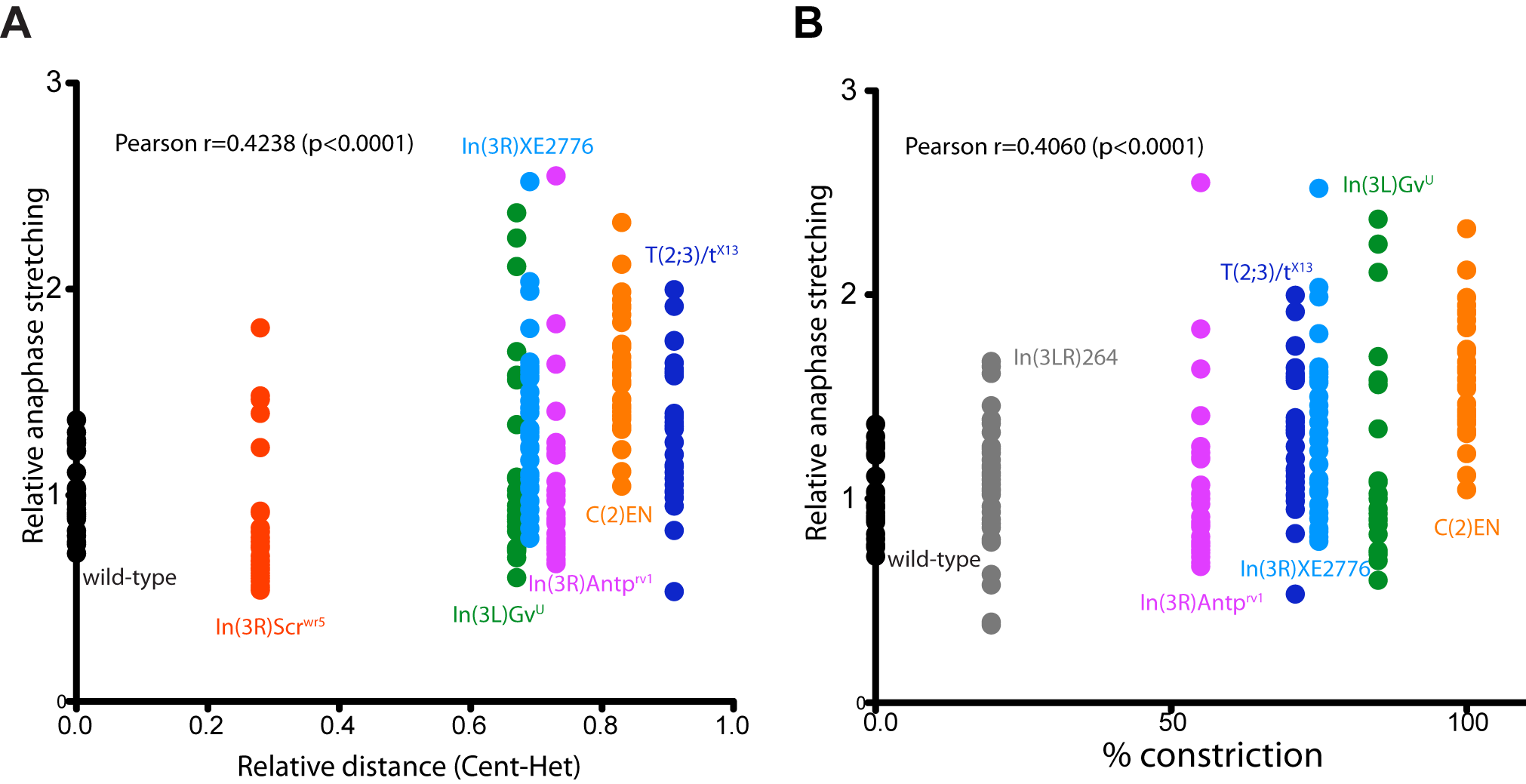

Supplement: Figure S9 — Correlation of anaphase chromatin stretching with the distance from the centromere (A) or the frequency of observed constriction (B). Datasets can be found in Table S2. (TIF) [file pbio.1001962.s009.tif]

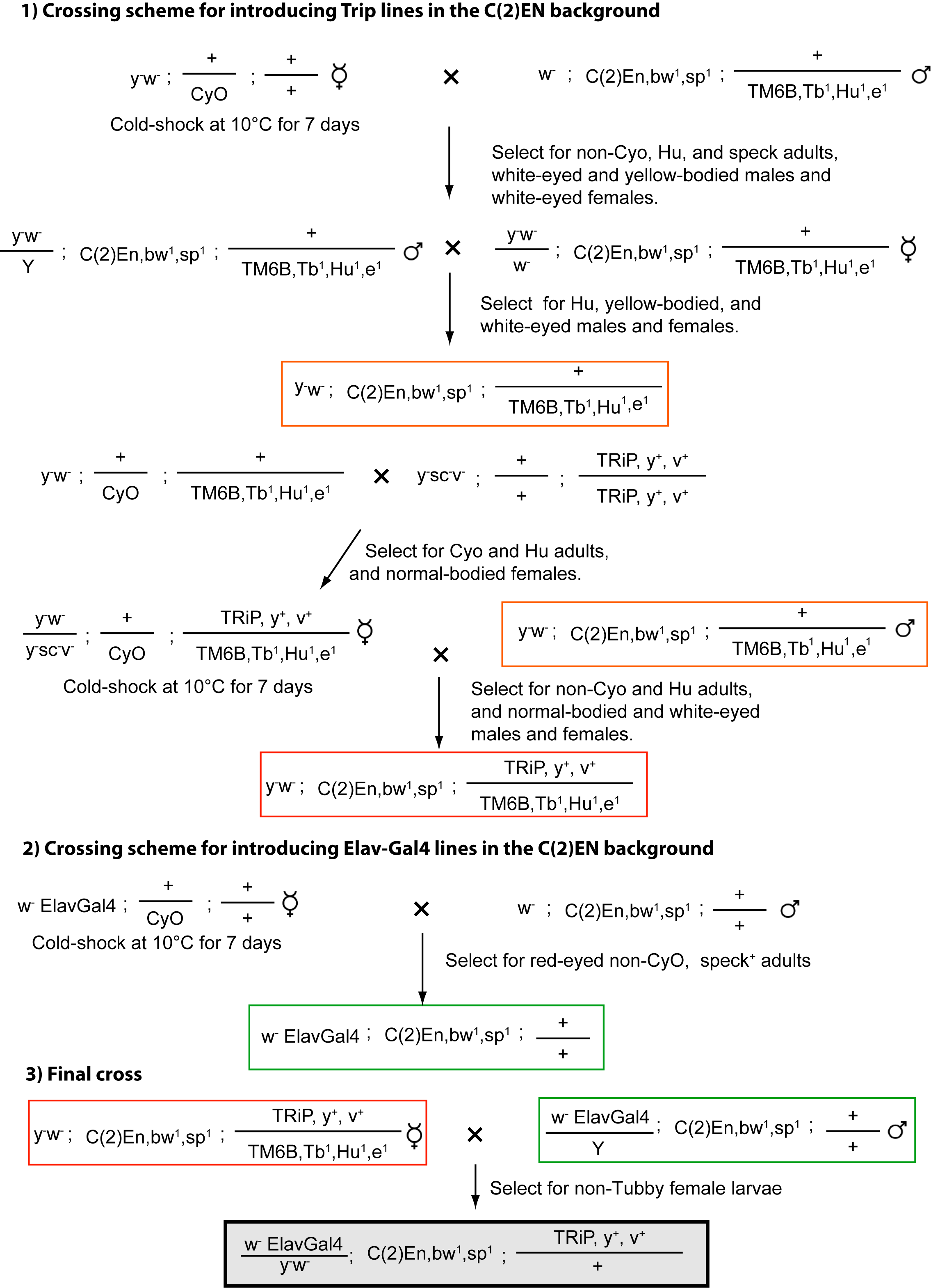

Supplement: Figure S10 — Schematic representation of the crossing strategy for RNAi experiments in the C(2)EN strain. (TIF) [file pbio.1001962.s010.tif]
