# Supplementary material for: Centromere-Independent Accumulation of Cohesin at Ectopic Heterochromatin Sites Induces Chromosome Stretching during Anaphase
Source: PLoS Biol. 2014 Oct 7;12(10):e1001962. doi: 10.1371/journal.pbio.1001962 (PMC4188515; doi:10.1371/journal.pbio.1001962)
Supplement: Table S1 — List of Drosophila stocks used in this study. (PDF) [file pbio.1001962.s011.pdf]

Table S1 - Drosophila stocks used in this study

**Genotype**	**Reference**
w;;tubpr-Rad21(wt)-EGFP 2, Rad21^{ex15} (rec 2.21)	This study
w;H2Av-mRFP;tubpr-Rad21(wt)-EGFP 2, Rad21^{ex15} (rec 2.21)	This study
w; C(2)En, bw^{1},sp^{1}; tubpr-Rad21(wt)-EGFP 2, Rad21^{ex15}/(TM6,C)	This study
w;P[w+, gCRC]II.1, P[w+, gCRC]II.2/CyO;	Schuh et al, 2007
w;HisH2AvD-mRFP1 II.2;	Schuh et al, 2007
w;P[w+, gCRC]II.1, P[w+, gCRC]II.2/CyO; tubpr-Rad21(wt)-EGFP 2, Rad21^{ex15} (rec 2.21)	This study
RNAi Nipped-B	TRiP #32406
RNAi SA	TRiP #33395
Elav-Gal4	Lin and Goodman, 1994
wapl^{C204}/FM7;;	Verni et al, 2000
wapl^{C204}/FM7i,$_{Actin-GFP}$;;Rad21$^{550-3TEV}$-EGFP, Rad21^{ex15}, pPolyubiquitin-H2A-mRFP1/TM3	Eichinger et al, 2013
yw; chip-NippedB-GFP (II);	Gause et al, 2010
w;C(2)EN, bw^{1},sp^{1};tubpr-Rad21(wt)-EGFP 2, Rad21ex15 (rec 3.1.3)	This study
w;C(2)EN, bw^{1},sp^{1};HisH2AvD-mRFP1 III.1	Martins et al, 2013
w^{1118}; P[GFP-HP1]3/TM6B, Tb^{1}	Bloomington #30561
w;C(2)EN, bw^{1},sp^{1};P[GFP-HP1]3	This study
T(2,3)lt^{X13}	Wakimoto and Hearn, 1990
In(3LR)264, mv1/TM6B, Tb1	Bloomington #1222
In(1)pdf, w^{a} pdf^{1}	Bloomington #980
In(3LR)pcv, pcv^{1}	Bloomington #6176
In(3R)Scr^{Wrv5}, red^{1} Scr^{Wrv5} e^{1}/TM3, Sb^{1}	Bloomington #2010
In(3R)Antp^{rv1}, osa^{Arv1}/TM3, Sb^{1}	Bloomington #1805
In(3L)gv^{U}, ru^{1} h^{1} gv^{U} eyg^{U} gl^{2} e^{4}/TM3, Sb^{1} Ser^{1}	Bloomington #1325
In(2R)XE-2776, PTP-ER$^{XE-2776}$/CyO, P[sevRas1.V12]FK1	Bloomington #5765
